# Facile Uptake and Release of Ammonia by Nickel Halide Ammines

**DOI:** 10.1002/cssc.201600140

**Published:** 2016-05-03

**Authors:** Joachim Breternitz, Yury E. Vilk, Elsa Giraud, Hazel Reardon, Tuan K. A. Hoang, Agata Godula‐Jopek, Duncan H. Gregory

**Affiliations:** ^1^ WestCHEM, School of ChemistryUniversity of GlasgowGlasgowG12 8QQUK; ^2^ Department of ChemistryLudwig-Maximilians Universität MünchenButenandtstraße 5-1381377MunichGermany; ^3^ Faculté des SciencesUniversité d'OrléansRue de Chartres45067Orleans Cedex 2France; ^4^ Airbus Group Innovations, TX681663MunichGermany; ^5^ Institute of Chemical EngineeringPolish Academy of Sciences44100GliwicePoland

**Keywords:** halides, nickel, structure elucidation, solid-state reactions, x-ray diffraction

## Abstract

Although major difficulties are experienced for hydrogen‐ storage materials to meet performance requirements for mobile applications, alternative fuel cell feedstocks such as ammonia can be stored in the solid state safely at high capacity. We herein describe the NiX_2_‐NH_3_ (X=Cl, Br, I) systems and demonstrate their exceptional suitability for NH_3_ storage (up to 43 wt % NH_3_ with desorption that begins at 400 K). The structural effects that result from the uptake of NH_3_ were studied by powder X‐ray diffraction (PXD), FTIR spectroscopy and SEM. NH_3_ release at elevated temperatures was followed by in situ PXD. The cycling capabilities and air stability of the systems were also explored. NH_3_ is released from the hexaammines in a three‐step process to yield the diammine, monoammine and NiX_2_ dihalides respectively and (re)ammoniation occurs readily at room temperature. The hexaammines do not react with air after several hours of exposure.

## Introduction

The current dilemma that faces designers of hydrogen‐storage materials is to balance a high gravimetric and volumetric hydrogen density with facile and reversible uptake and release.^1^ Additionally, such a material would need to be inexpensive and safe. Whereas some metal and complex hydrides can store large quantities of hydrogen, the extraction of the gas from the solid (reversibly) is not possible under the typical operating conditions of polymer electrolyte membrane (PEM) fuel cells; the interaction between the “host” and the chemically bound H is enthalpically too strong.^2^ In contrast, readily reversible materials based on physical H absorption, such as metal– organic or covalent organic frameworks (MOFs or COFs, respectively) typically exhibit low hydrogen capacities under ambient (or above ambient) temperature.^3^, ^4^ A state‐of‐the‐art framework material for hydrogen storage such as MOF 177, for instance, has a hydrogen storage capacity of 7.5 wt % at 77 K and 70 bar, whereas the storage capacity at ambient pressure is significantly lower (1 wt %).^5^ In such cases, the interaction between the host and H is enthalpically too weak for storage at ambient pressure.

One approach to the significant challenges associated with the storage of hydrogen, especially for mobile applications, is to consider “indirect sources” of hydrogen or the complete replacement of hydrogen with an alternative high‐energy‐density sustainable fuel. Ammonia is one such possibility with the capacity to be cracked catalytically to hydrogen and nitrogen or, as it has an energy density of 13.6 GJ m^−3^ (at 10 bar),^6^ to be used as a fuel in its own right. This task is made easier by the emergence of efficient direct ammonia fuel cells (DAFCs) that would potentially permit on‐board storage and power generation within the working temperatures of the system.^7^

Ammonia contains 17.6 wt % H_2_ and has the advantage of an existing production and distribution infrastructure. Its current inexpensive (but unsustainable) production in the Haber–Bosch process makes it a cheap alternative to the direct use of hydrogen and alternative sustainable methods of generation offer hope for future “greener” large‐scale production.^8^, ^9^, ^10^, ^11^, ^12^ In principle, ammonia could be stored as a gas or liquid and utilised directly from a tank for mobile applications. Indeed, ammonia molecules are much less mobile and far less able to diffuse through the walls of a container than hydrogen, which renders the escape (loss) of fuel unlikely. Further, unlike hydrogen, ammonia does not form explosive mixtures with air readily. However, ammonia is toxic and corrosive (although its characteristic strong odour enables rapid leak detection) and demands safe and secure storage, especially in transit. High‐ capacity solid‐state NH_3_‐storage materials offer a potentially satisfactory solution.

Solids that contain ammonia or its salts have a long history of chemical use that dates back to Arabic alchemists, and the first European Sal Ammoniac factory was established in Edinburgh as early as 1765.^13^ Moreover, the often colourful transition metal complex solutions (known as ammines) have attracted the attention of generations of chemists.^14^ In fact, ammine complexes offer the possibility to store NH_3_ chemically in the solid state. Alkaline earth metal halides are particularly suitable, and the highest reversible gravimetric capacities achievable (safely) are for the MgCl_2_‐NH_3_ system (51.8 wt % for [Mg(NH_3_)_6_]Cl_2_).^15^ Changing the metal centre offers the possibility to tailor the thermodynamics and kinetics of ammonia sorption, and transition metal halides are in many ways chemically ideal candidates to form NH_3_‐storage materials. They combine a strongly salt‐like character, which is important to form complex cations, with the affinity of transition metals towards ammonia as a ligand to form ammine coordination complexes.^16^, ^17^

We describe here a systematic study of the ammonia uptake and release behaviour of the nickel(II) halides, NiX_2_ (X=Cl, Br, I). We used a combination of powder X‐ray diffraction (PXD) and spectroscopy to confirm the structures of each of the respective halide hexa‐ and diammines. We combined these data with those from thermal analysis to determine the decomposition conditions and reaction pathways in each halide system from the fully ammoniated compounds to the respective dihalides.

## Results and Discussion

It is perhaps slightly unexpected to observe how readily the nickel halides react with dry ammonia at room temperature. By way of example for NiCl_2_, over 50 % of the total theoretical ammonia uptake (44.0 wt %) occurred within 5 min, and >90 % of the gravimetric capacity was achieved in ≈25 min under 1 bar NH_3_ (Figure S1). After 120 min, the chloride absorbed 42.9 wt % NH_3_ (98 % of the theoretical maximum). The uptake thus compares favourably with other metal ammines and also the best porous materials under similar conditions.^17^, ^18^ The solids increased in volume dramatically on gas uptake and evolved heat in the process. The respective solids also changed colour (NiCl_2_: yellow, NiBr_2_: brown, NiI_2_: black) to pale violet (for NiCl_2_ and NiBr_2_) or white (NiI_2_) as a result of ammoniation. Microanalyses of the different products confirmed that the hydrogen composition of the powders matched that expected for the stoichiometric hexaammine halides (H [wt %] for [Ni(NH_3_)_6_]X_2_: X=Cl: found: 7.7(7), calcd: 7.77; X=Br: found: 5.5(3), calcd: 5.62; X=I: found: 4.4(3), calcd: 4.33).

The FTIR spectra of the ammoniated halides were ostensibly very similar and are in good agreement with previous studies of ammines (Table S1 and Figure S2).^19^, ^20^ On one level, this is not surprising because the complex anions are isolated species in the solid state so the ligand vibration modes are largely independent of the interactions from the extended structure. Nevertheless, the N−H modes can provide valuable information with regard to the strength of the Ni−N bond and the existence and relative strength of H⋅⋅⋅X hydrogen bonds.^21^ The N−H stretching band positions are effectively unchanged with X. There is a tendency towards a shift of bands to lower wavenumbers (most pronounced for the rocking mode, *ρ*) across the series from X=Cl through Br to I with the single exception of the symmetrical deformation mode. It has been noted previously that the *δ*_s_ mode is coupled strongly to the deformation of the H−N−Ni bond angle.^20^ The upward shift in the *δ*_s_ frequency from X=Cl through Br to I implies a decrease of the Ni−N bond strength (such an effect is even clearer for series of complexes in which the central metal is varied).^22^ Conversely, the decrease in the NH_3_ rocking mode frequency from X=Cl through Br to I is symptomatic of progressively weaker H⋅⋅⋅X hydrogen bonds.^22^ Otherwise, broadly the IR analysis of the different compounds indicates that the N−H bonds are weakened compared to that of gaseous ammonia and, therefore, it would be expected that a combination of M−N bonds and H⋅⋅⋅X interactions become significant to weaken the N−H bonding in the solid state. The relative individual contributions of the two interactions provide a further motivation for a detailed investigation of the crystal structures as discussed below.

**Figure 1:**
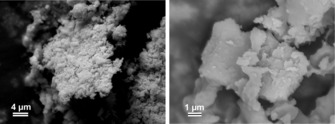
SEM images of [Ni(NH_3_)_6_]I_2_ samples produced by ammoniation at room temperature.

Each of the ammines were pale in colour, and the white colour of [Ni(NH_3_)_6_]I_2_ is perhaps the most unexpected. Although ammonia uptake resulted in notable volume changes for all of the halides, for the iodide in particular, the colour change was accompanied by a dramatic volume increase upon reaction. A combination of evidence from SEM imaging and peak broadening in the PXD patterns reveals very small crystallite sizes in the hexaammine powders. The size distribution in the samples is far from monodisperse, and varies from sub‐micron crystallites (which measure hundreds of nanometres or less) to agglomerations of particles in the micron range, as shown by SEM imaging (Figure 1, Figures S8 and S9). A corroborative quantitative assessment of the crystallite dimensions and size distribution could not be made using either diffraction data by the Scherrer equation or BET adsorption. The former applies only to monodisperse samples of isotropic particles, and the latter is practically challenging given the possible release of NH_3_ gas under high vacuum. The size distribution is undoubtedly related to the strong exothermic driving force of the reaction, and we hope to correlate the uptake reaction kinetics with the dihalide and ammine particle sizes in future experiments. Energy‐dispersive X‐ray (EDX) analysis of the ammines gave a Ni/X elemental ratio of approximately 1:2 as expected (for [Ni(NH_3_)_6_]I_2_, Ni/I=1:1.9(1)) and confirmed the absence of any impurities. Nitrogen was detectable by EDX methods but could not be quantitatively determined reliably.

**Figure 2:**
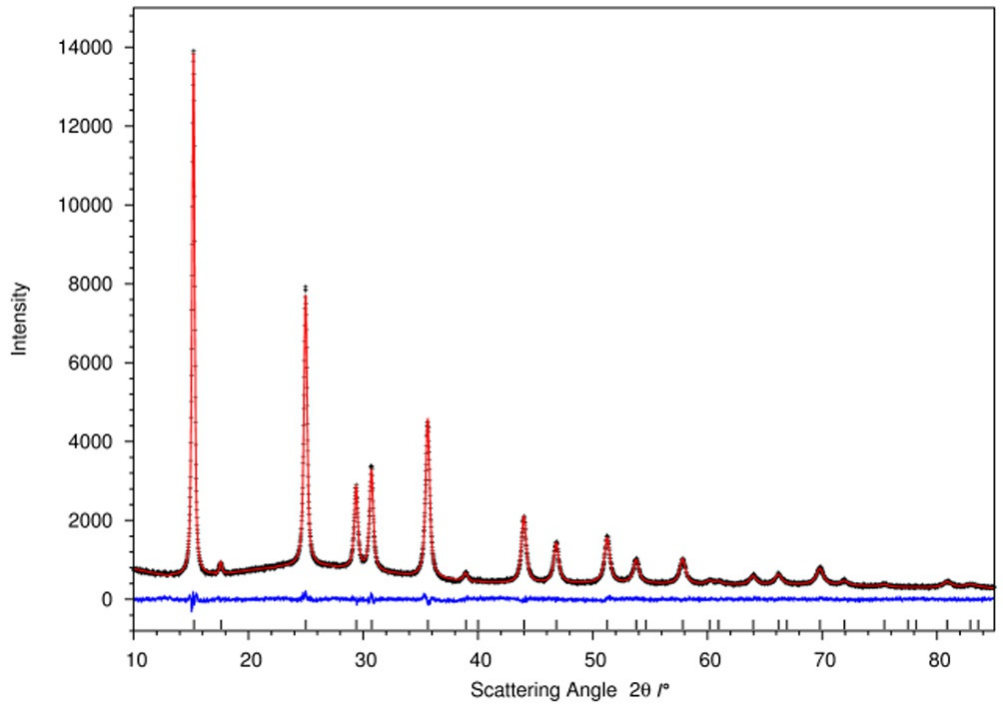
Rietveld refinement profile for [Ni(NH_3_)_6_]Cl_2_. The red line represents the calculated pattern, the black crosses show the measured pattern and the difference is represented by the blue line. Reflection positions are indicated by black ticks.

**Figure 3:**
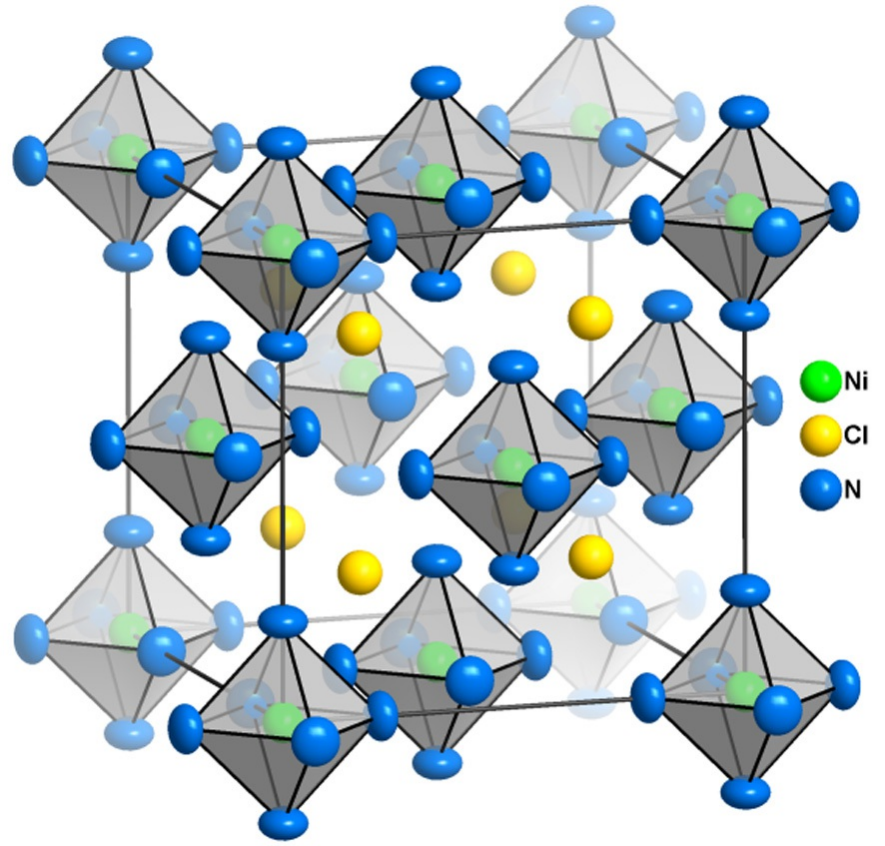
Representation of the structure of [Ni(NH_3_)_6_]X_2_ (shown here for X=Cl). The atom ellipsoids represent 90 % spatial probability. Octahedra are centred by Ni. H atoms are omitted in all but the lower right octahedron for clarity.

A comparison of the PXD patterns of the reaction products with documented crystal structures of the corresponding [Ni(NH_3_)_6_]X_2_ crystals using POWDERCELL confirmed the identity of the ammoniated compounds.^23^, ^24^ The patterns were first refined using a structure‐less Le Bail fitting in which all refined profile parameters were allowed to converge to extract reflection intensities for the subsequent structure solution. The automated structure solution with Superflip allowed the location of all non‐hydrogen atoms in positions according to structures reported previously. The obtained structures were then refined using Jana2006 (Figures 2 and 2, 3 for X=Cl, detailed analysis for X=Br, I is given in the Supporting Information, Table 1) using the profile obtained in the Le Bail fitting.^25^, ^26^ The profile parameters were refined simultaneously in the later stages of the refinements. (Details of the refinements can be found in the Supporting Information). This procedure yielded good refinement stability together with rapid convergence.

**Table 1:** Rietveld Refinement results for [Ni(NH_3_)_6_]X_2_.

Formula	[Ni(NH_3_)_6_]Cl_2_	[Ni(NH_3_)_6_]Br_2_	[Ni(NH_3_)_6_]I_2_
Crystal system	cubic	cubic	cubic
Space group	*Fm* $\bar{3}$ *m*	*Fm* $\bar{3}$ *m*	*Fm* $\bar{3}$ *m*
*a* [Å]	10.0744(3)	10.3826(3)	10.8999(2)
Volume [Å^3^]	1022.49(6)	1119.22(6)	1295.00(3)
*Z*	4	4	4
Calculated density, *ρ*_x_ [g cm^−3^]	1.506	1.903	2.13
*R*_p_; *wR*_p_	0.036; 0.046	0.041; 0.054	0.034; 0.044
Goof; *χ* ^*2*^	1.44; 2.07	1.80; 3.24	1.26; 1.59
*R*_obs_; *wR*_2_(all)	0.030; 0.044	0.019; 0.022	0.026; 0.029
*d*(Ni−N) [Å]	2.155(2)	2.152(2)	2.172(2)

The fitting of hydrogen positions in these compounds is not straightforward as the electron density is nearly uniformly distributed in a circle around the triangular molecular positions.^27^ It nevertheless proved crucial to add the hydrogen atoms; only a model that included all the theoretical electron density was able to yield accurate Ni−N bond lengths. The hydrogen atoms contribute 30 % of the total electron count and shift the centre of the electron density considerably further from the Ni position if not treated appropriately. We chose a description of the ammonia groups in which the three H atoms and the ligated Ni atom form the vertices of a N‐centred tetrahedron. The position of the H atoms was set automatically by the refinement program to lead to a situation in which three hydrogen positions are heavily distorted because of the clash of the three‐fold molecular symmetry and the four‐fold screw axis in the crystal structure. The hydrogen atoms were, therefore, set to an occupation of 1/8 on each (*x*, *y*, *z*; 192 *l*) site. Whereas realistic N−H bond lengths would be expected to be ≈1.01 Å, as found in gaseous or solid ammonia,^28^, ^29^ the apparent N−H distances were set to 0.87 Å as suggested by Jana2006. Bonds to hydrogen appear short in X‐ray diffraction (XRD) because of the strong polarisation of the solitary electron.^30^ Hydrogen bonding, N−H⋅⋅⋅Cl would invoke the four isotropic Cl atoms that surround the circle of hydrogen positions. If we consider the position of any nominal Cl atom, the H atoms within an angular range of ±45° around the shortest possible Cl−H distance are closest to this Cl atom as opposed to the other Cl atoms (Figure S26 a). To estimate which of the H⋅⋅⋅Cl distances can be considered to be hydrogen bonds, the bond valence parameters based on Pauling's considerations of ionic crystals were used for comparison (Figure S26 b).^31^ The bond valence parameter is a robust, semi‐quantitative estimation of bond strength based on the distance between the atoms. Typical hydrogen bonding bond–valence parameters for H⋅⋅⋅Cl are between 1 and 10 % of the value for a single equivalent covalent bond, which corresponds to a bond valence parameter of 1.^32^ Therefore, H⋅⋅⋅Cl distances below ∼3 Å can be considered as short enough to contribute significantly as hydrogen bonds (Figure S26 b). However, as noted above, the hydrogen positions are shifted systematically towards the N atom in the derived model. Each H, therefore, appears to be more distant from the Cl atom, and the H⋅⋅⋅Cl distance is consequently longer than that in reality. One also has to be mindful that a static approximation cannot be entirely valid given the dynamic disorder of the ammonia ligands (rotation around the Ni−N bonding axis). Hence, hydrogen bonding could be observed in *any* rotational position because of the symmetrical arrangement of the four chloride anions around any ammonia molecule (Figure S24). This leads to the absence of an energetic minimum of any significance for any rotational arrangement (i.e., one H atom at the ideal shortest distance would extend the other H⋅⋅⋅Cl distances unavoidably and vice versa). The arrangement of the Cl atoms thus leads to a low energetic rotation barrier for the ammonia ligands. A definitive model for the hydrogen disorder and a more complete description of the hydrogen bonding network is only likely to be forthcoming from powder neutron diffraction experiments with deuterated materials, and we expect to report the outcome of such experiments in due course.

The structures of [Ni(NH_3_)_6_]X_2_ (X=Cl, Br, I) are built from isolated complex [Ni(NH_3_)_6_]^2+^ cations and halide anions (Table 1 and Supporting Information). The complex cations are defined principally by the Ni−N distance. As might be expected, this is rather similar for the different complexes (Cl: 2.155(2) Å, Br: 2.152(2) Å, I: 2.172(2) Å). Although the distances for the chloride and the bromide compounds are equal within error, the distance in the iodide analogue is slightly longer, which indicates a general trend for weaker metal−ligand bonds for the heavier halide anions. This finding corroborates the observed increase in the *δ*_s_ FTIR band frequency. Although, in reality, the constraint imposed on the N−H bond length leads to an over‐estimation of the X⋅⋅⋅H distances, nevertheless the distances from the respective halide anions to the hydrogen atoms increase in the series (H−Cl: 2.75 Å, H−Br: 2.85 Å, H−I: 3.05 Å; anionic radii: *r*(Cl^−^)=1.67 Å, *r*(Br^−^)=1.82 Å, *r*(I^−^)=2.06 Å^33^) These increases are consistent with the decrease in the frequency of the *ρ*(NH_3_) band in the FTIR spectra and weaker hydrogen bonding interactions from N−H⋅⋅⋅Cl to N−H⋅⋅⋅I. The long distance between each anion and the central atom of the cationic complex (e.g., 4.36 Å for X=Cl) confirms that the interaction is essentially electrostatic. Although this might not be unexpected for the packing of charged transition metal complexes and counter anions in the solid state, what is perhaps more surprising is the degree of structural rearrangement that occurs upon ammoniation under mild conditions. The starting (de‐ammoniated) materials NiX_2_ (CdCl_2_‐type, *R* $\bar{3}$ *m*) are composed of layers of edge‐connected [NiX_6_] octahedra in which a strong central atom–ligand (metal cation–anion) interaction exists (e.g., Ni−Cl=2.43 Å),^34^ and hence the bonding environment changes dramatically during the exothermic ammoniation reaction to form the ammines.

**Figure 4:**
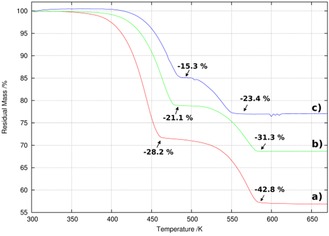
TGA profiles of [Ni(NH_3_)_6_]X_2_ in which X is a) Cl, b) Br and c) I. The observed mass loss steps are given as relative values.

**Figure 5:**
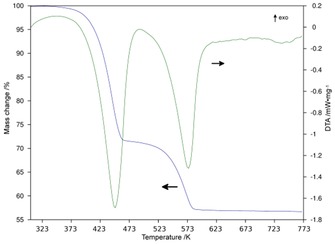
TG‐DTA profile for [Ni(NH_3_)_6_]Cl_2_ with TG (blue) and DTA (green).

**Figure 6:**
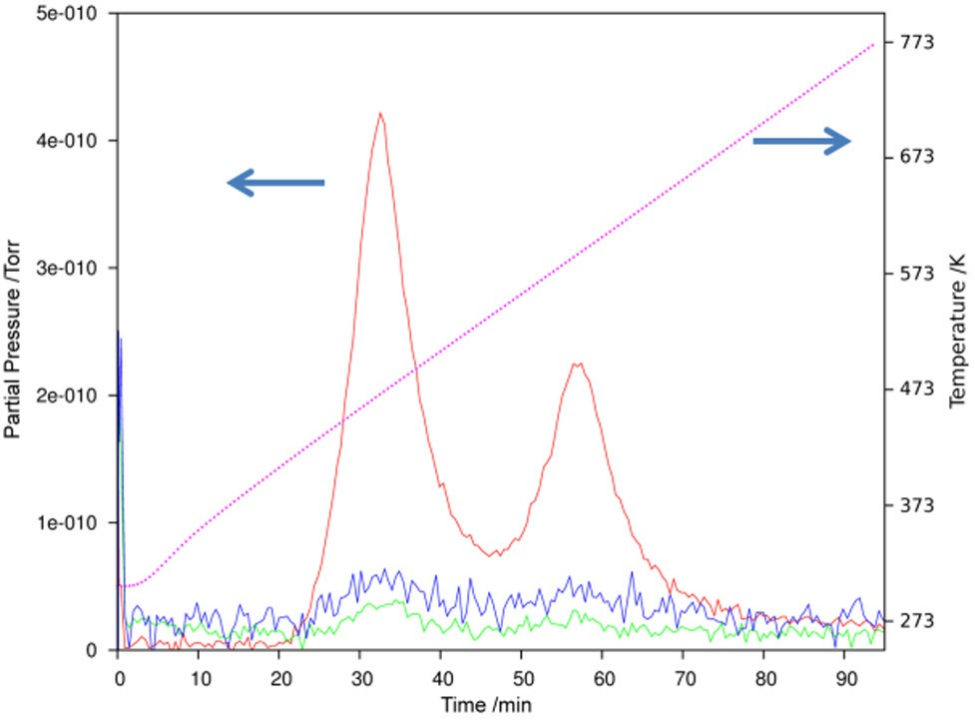
Partial MS spectrum for the evolved gas from the TG‐DTA experiment for [Ni(NH_3_)_6_]Cl_2_ (Figure 5) with *m*/*z*=2 (H_2_^+^, blue), 17 (NH_3_^+^, red) and 28 (N_2_^+^, green). The TG‐DTA temperature profile is given by the dotted line (magenta).

Several studies have examined the thermal desorption of one or more of the nickel hexaammines previously but results have been apparently contradictory in terms of the number and nature of decomposition steps observed.^24^, ^35^, ^36^ Further, although the structures of the diammines Ni(NH_3_)_2_X_2_ (X=Cl, Br) have been reported, the details of the changes in phase/structure with temperature for the NiX_2_‐NH_3_ systems are not comprehensive and the structure of the iodide diammine remains as yet uncharacterised.^37^ Thermogravimetric and differential thermal analysis with mass spectrometry (TG‐DTA–MS) with a heating rate of 5 K min^−1^ (Figure 4) yielded the data listed in Table S3 and further demonstrated that NH_3_ was the only gas evolved in significant amounts in the thermal decomposition of all the [Ni(NH_3_)_6_]X_2_ samples (Figures 5 and 6 for X=Cl, Figures S4–S7 for X=Br, I).

From the TG profiles, ammonia release appears to proceed in two steps. The onset temperatures for the first desorption step lie in the range from close to room temperature (≈303 K) for [Ni(NH_3_)_6_]Cl_2_ to approximately 353 K for both [Ni(NH_3_)_6_]Br_2_ and [Ni(NH_3_)_6_]I_2_. In all cases de‐ammoniation is complete below 600 K, and the final reaction step to the respective expected dihalides finishes at progressively lower temperatures from X=Cl (580 K) to X=I (549 K). The MS spectra taken from the evolved gas (Figure 6 and Supporting Information) show the desorption of NH_3_ with some simultaneous detection of N_2_ and H_2_ (approximately one order of magnitude lower in terms of partial pressure) in each case. Given that a solid oxide direct ammonia fuel cell, for example, would crack ammonia to N_2_ and H_2_ at the anode, neither of these decomposition products would present any issues from their presence in the feed gas stream. The mass spectra show two peaks that appear to correspond to a two‐step desorption process with Ni(NH_3_)_2_X_2_ as the only apparent intermediate compound for each X. Accordingly, the DTA signal shows two endothermic peaks synchronous to the two weight‐loss steps. We saw no evidence under these conditions (i.e., at a heating rate of 5 K min^−1^ or indeed at a slower heating rate of 2 K min^−1^) to support a de‐ammoniation step to the equivalent monoammines from the TG, DTA, d(TG)/d*T* or the MS profiles for any of the halide diammines.

**Figure 7:**
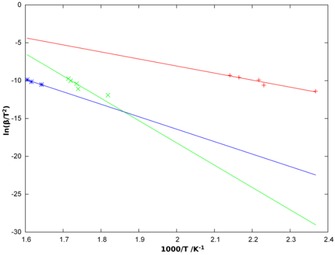
Kissinger plots for the three steps in the decomposition of [Ni(NH_3_)_6_]Cl_2_ with the hexaammine → diammine step in red (+), the diammine → monoammine step in green (×) and the monoammine → dihalide decomposition in blue (*). The linear fit is shown as a solid line in each case.

Given that a further de‐ammoniation step (to the monoammine) has been described previously and that the monoammines Ni(NH_3_)X_2_ (X=Cl, Br) are well known (and can be synthesised by the solid‐state reaction of NiX_2_ with Ni(NH_3_)_2_X_2_, for example) and have been structurally characterised,^38^ we performed a further study of the thermal decomposition of [Ni(NH_3_)_6_]Cl_2_ at variable heating rates. We were thus able to derive the activation energy for each of the decomposition steps by applying the Kissinger method.^39^ The resulting activation energies (Figure 7, Table S37) are 76±1, 245±3 and 137±3 kJ mol^−1^ for the decomposition of the hexaammine, diammine and monoammine, respectively.

Hence TG‐DTA‐MS experiments conducted at higher heating rates (15 and 20 K min^−1^) provide vital evidence for the formation of an intermediate monoammine before the decomposition to NiX_2_. Given that the monoammine decomposition step has a lower activation energy than the equivalent diammine ammonia loss, the latter process is effectively rate‐determining. At 15 and 20 K min^−1^, the rate of the diammine decomposition becomes comparable to that of the monoammine, and the complete three‐step decomposition mechanism is, therefore, observed. These observations concur with the three‐step mechanism observed by George and Wendtland^36^ under heating at rates above 5 K min^−1^. Hence, overall one can conclude that the de‐ammoniation of [Ni(NH_3_)_6_]X_2_ for X=Cl follows the three‐step process shown in Equations 1–(1).

(1) $$\left[\mathrm{Ni}\left(\mathrm{NH}{}_{3}\right){}_{6}\right]\mathrm{Cl}{}_{2}\overset{-4\;\mathrm{NH}{}_{3}}{\underset{}{\to }}\mathrm{Ni}\left(\mathrm{NH}{}_{3}\right){}_{2}\mathrm{Cl}{}_{2}$$

(2) $$\mathrm{Ni}\left(\mathrm{NH}{}_{3}\right){}_{2}\mathrm{Cl}{}_{2}\overset{-\mathrm{NH}{}_{3}}{\underset{}{\to }}\mathrm{Ni}\left(\mathrm{NH}{}_{3}\right)\mathrm{Cl}{}_{2}$$

(3) $$\mathrm{Ni}\left(\mathrm{NH}{}_{3}\right)\mathrm{Cl}{}_{2}\overset{-\mathrm{NH}{}_{3}}{\underset{}{\to }}\mathrm{NiCl}{}_{2}$$

The observed weight losses are in very close agreement with those expected for each of the above de‐ammoniation steps (Table 2). To elucidate the nature of the phase transitions indicated by thermal analysis and to ascertain the identity of the intermediate diammine phases, we performed in situ variable‐temperature PXD experiments.

**Table 2:** Thermal decomposition (STA) results for [Ni(NH_3_)_6_]Cl_2_ with a heating ramp of 20 K min^−1^.

*n*(NH_3_)	Mass changes obs. (calcd) [wt %]		*T*_TG(STA)_ [K]		*T*_peak_ [K]
final	total	stepwise	onset	final	DTA
2	27.5 (29.4)	27.5 (29.4)	415.6	470.6	467.1
1	35.1 (36.7)	7.5 (7.3)	560.2	585.3	583.5
0	41.3 (44.0)	6.2 (7.3)	600.5	631.0	623.1

**Figure 8:**
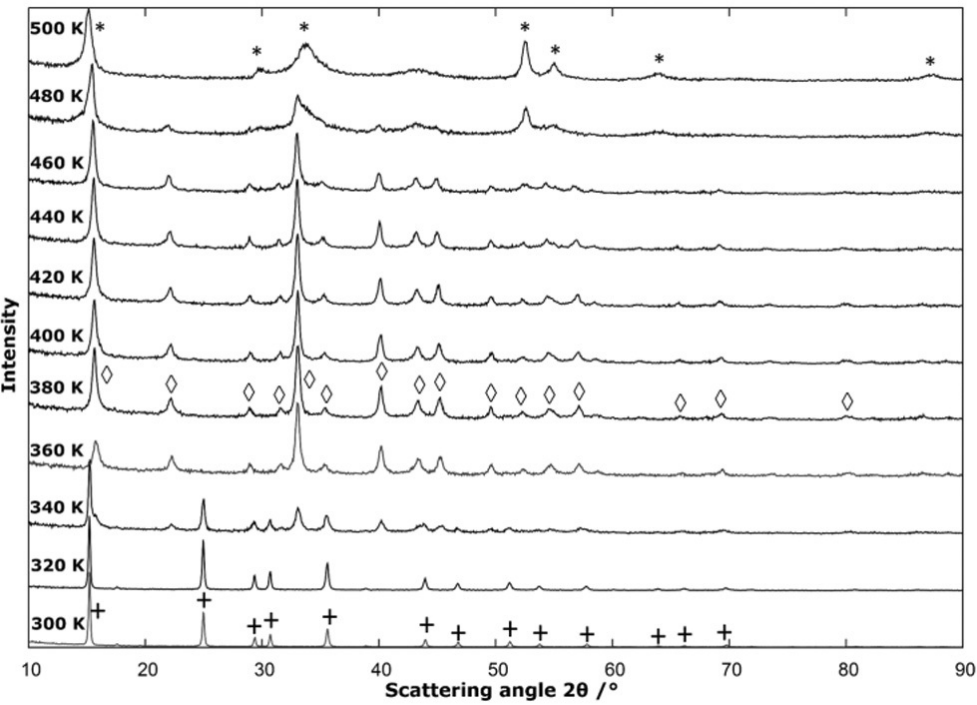
Variable‐temperature in situ PXD patterns of the thermal decomposition of [Ni(NH_3_)_6_]Cl_2_, which indicates the presence of the [Ni(NH_3_)_6_]Cl_2_ (+), Ni(NH_3_)_2_Cl_2_ (◊) and NiCl_2_ (*) phases.

As a result of the high desorption rates and the configuration of the diffraction experiment, the desorption steps are shifted to lower temperature values than those observed in the anisothermal thermal analyses. The chloride desorption is typical for all of the NiX_2_ ammines and will be discussed in more detail as an example (the in situ PXD patterns of the desorption of the other ammines is shown in Figures S21 and S22). The hexaammine phase observed at 298 K is completely transformed into the diammine phase at 360 K, which in turn decomposes gradually to NiCl_2_. The formation of the poorly crystalline NiCl_2_ phase can be observed from 460 K and is completed by 480 K (Figure 8). The intermediate monoammine phases could be not detected under the conditions of the diffraction experiments.

SEM images and FTIR spectra were also acquired for the intermediate diammines. The morphology (Figure S10) of the diammines is retained from the respective hexaammines and a broad particle size distribution is again prevalent. The IR spectra of the diammine samples (Figure S3) can all be assigned according to the bands expected from previous studies (Table S2).^40^ The band positions are broadly similar to those of the hexaammines with the notable exception of the very strong symmetric scissoring mode, the wavenumber of which is increased by over 50 cm^−1^. This is probably because of the more rigid complex environment in the diammine phase as the coordinating halides bridge the complexes and stabilise the octahedral environment within the 1∞ [Ni(NH_3_)_2_X_4/2_] chains (see below). This again is in accordance with the fact that the *δ*_s_ mode is the most sensitive to deformations of the Ni−N bond.^20^

In accordance with the results of Leineweber and Jacobs for nickel chloride and bromide diammines,^37^ we obtain Ni(NH_3_)_2_X_2_ (X=Cl, Br, I) as the β‐type polymorphs with the Cd(NH_3_)_2_Cl_2_ structure type (orthorhombic space group *Cmmm*), in which the metal atom is surrounded by octahedra made up of two *trans* NH_3_ ligands and four X atoms (see Supporting Information). The individual octahedra share edges that contain X atoms to form 1∞ [Ni(NH_3_)_2_X_4/2_] chains. Although the PXD pattern quality from the employed experimental configuration was not ideal, we were able to refine the structures for Ni(NH_3_)_2_X_2_ for X=Br, I. The refinement for X=Cl, however, did not reach convergence if the thermal displacement parameters were varied and permitted a structural model to be obtained only if the temperature factors were fixed to generic values. This is a facet of the relatively low quality of the powder data and the more uniform electron density distribution compared to that of the ammines that contained the electron‐rich Br^−^ and I^−^ anions. Therefore, the refined structure for Ni(NH_3_)_2_Cl_2_ should be viewed as a partial model.

Our results are in close agreement with the structural analyses made by Leineweber and Jacobs, and we are able to present the first full structural refinement for Ni(NH_3_)_2_I_2_. However, the distances between the central Ni atom and the surrounding ligands refined from our in situ data are considerably longer than the distances refined by Leineweber et al. (Table 3).^37^ This is almost certainly a consequence of the variation in measurement temperatures. Most notable is the strong elongation of the Ni−N bond, and the change in the Ni−Br distance is much less distinct compared to the literature data. That the elongation of the Ni−N bond is primarily a thermal expansion effect is manifested in the consistent distances for both the X=Br and I diammines (at 373 and 393 K, respectively).

**Table 3:** Cell parameters and inter‐atomic distances in the chains of Ni(NH_3_)_2_X_2_ (X=Br, I) obtained in situ compared with the results obtained by Leineweber et al.

X	*T*_meas_^[a]^ [K]	*a* [Å]	*b* [Å]	*c* [Å]	*d*(Ni−X) [Å]	*d*(Ni−N) [Å]
Br^37^	–	8.273	8.297	3.851	2.690(1)	2.091(6)
Br	373	8.298(2)	8.324(2)	3.8684(7)	2.716(3)	2.23(2)
I	393	8.726(3)	8.800(2)	4.142(1)	2.929(3)	2.23(3)

[a] Measurement temperature.

**Figure 9:**
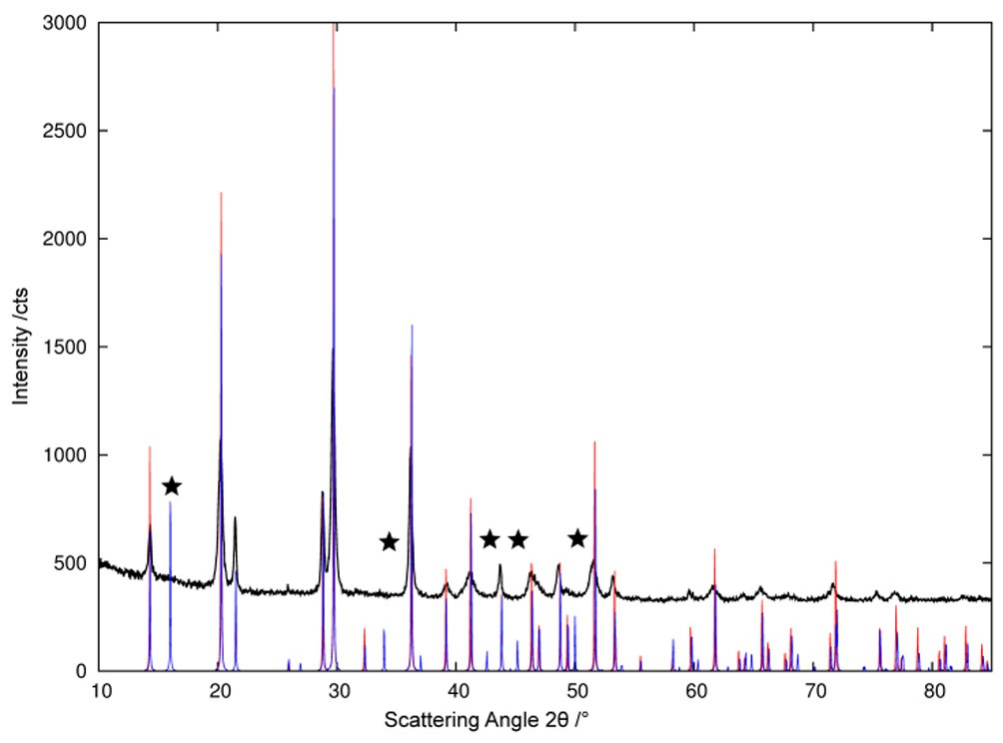
Comparison of the observed PXD pattern for Ni(NH_3_)_2_I_2_ at 393 K with the calculated reflection positions and relative intensities for the β‐type (red) and α‐type (blue) diammine structures. The strongest reflection positions exclusive to the α‐polymorph are marked with a star and are absent in the experimental pattern.

Notably, at no point did our in situ experiments show any evidence of a phase‐transition of the diammines into the α‐type structure (Mg(NH_3_)_2_Br_2_ type; orthorhombic space group *Pbam*), which is reported to form during annealing at 573 K.^37^ Although the α‐type structure exhibits a very similar diffraction pattern to the β‐type structure (Figure 9), the α‐polymorph generates additional reflections that would be easily detectable if present. The diffraction evidence would imply that the α‐type structure is only likely obtained under a higher ammonia pressure as the diammines would otherwise decompose below 573 K (as evidenced by our thermal analysis data).

The two diammine structure types are closely related to each other and only differ significantly through the packing of the 1∞ [Ni(NH_3_)_2_X_4/2_] chains. Half of the chains in the α‐type are rotated by 90° compared to that of the β‐type. The inter‐chain relationships are different in the two structure types. If we consider the Ni(NH_3_)_2_Br_2_ polymorphs as examples, although each chain in the β‐type structure is surrounded by four nearest‐neighbour chains (*d*(Ni−Ni)=5.8584 Å), the chains in the α‐type have only two nearest neighbours (*d*(Ni−Ni)=5.8650 Å).^37^ However, the next‐nearest neighbours in the α‐type are much closer (four chains with *d*(Ni−Ni)=6.5541 Å) than those in the β‐type (two chains with *d*(Ni−Ni)=8.2730 Å and two chains with *d*(Ni−Ni)=8.2970 Å), which stabilises the α‐type over the β‐type structure at higher temperatures. This is also consistent with the hydrogen bonding situation and the lower density of the α‐type that overall underlines the fact that the α‐phase is the thermodynamically disfavoured phase. The relationships between the stability of the phases as a function of ammonia partial pressure will be the subject of further studies. The NiX_2_ phases formed upon further heating are poorly crystalline under the synthesis conditions employed here. The levels of crystallinity were comparable with those observed in diffraction patterns for the equivalent halide starting materials supplied commercially.

Sørensen and co‐workers have established a model to explain the absorption–desorption mechanism for similar metal dihalide ammonia reactions by applying a model of chain abstraction from the surface.^17^ The apparent decrease in the observed crystallite size and bulk density that we observe during the gas–solid phase formation of the hexaammines is consistent with the postulation of a surface reaction that leads to the flaking/exfoliation of [Ni(NH_3_)_6_]X_2_ product from the surface of the dihalide starting material. Conversely, however, the hexaammine desorption reaction would appear to be consistent with a transformation process that invokes isolated [Ni(NH_3_)_6_]^2+^ complex cations and halide anions rather than extended 1∞ [Ni(NH_3_)_6_]X_2_} chains in which the individual component octahedra are separated from each other by significant distances (edge‐to‐edge distance 4.099(2) Å for X=Cl).^17^ Further, in the chloride hexaammine, for example, the anions are 2.520(2) Å away from the {2 0 0} planes that connect neighbouring [Ni(NH_3_)_6_] octahedral edges and 3.128(2) Å away from the nearest [Ni(NH_3_)_6_] octahedron face. Given the distances involved and the implications with regard to the strength of the chain, it might seem unlikely that the reaction takes place by the combination of these hypothetical 1∞ [Ni(NH_3_)_6_]X_2_} species. Alternatively, one might conceive of a model in which the metal–ligand recombination during the hexaammine to diammine ammonia desorption is rather governed by filling vacancies in the complex octahedra left by desorbing ammonia. The only possibility to fill these vacancies is by the approach and incorporation of the surrounding halide anions into the ligand coordination sphere. The so‐induced distortion in the ligand sphere could then provoke the subsequent loss of further ammonia molecules and their replacement by chloride anions in the manner of a self‐catalysing reaction until the stable 1∞ [Ni(NH_3_)_2_Cl_4/2_] configuration is reached.

**Figure 10:**
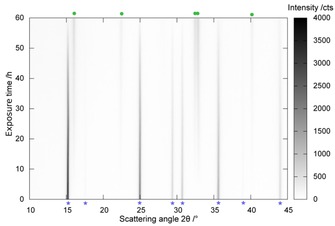
Exposure of [Ni(NH_3_)_6_]Cl_2_ to air under ambient conditions followed by time‐resolved PXD. The blue stars (bottom) mark [Ni(NH_3_)_6_]Cl_2_ and the green circles (top) the phase formed following air exposure.

An important characteristic of potential fuel storage materials in terms of safety is their chemical stability in air. We were able to demonstrate recently that ligated water in nickel complex materials can be replaced easily by ammonia.^41^ There are clear practical advantages if the storage material is robust in air over a reasonable timescale. We have followed the potential reactivity of [Ni(NH_3_)_6_]Cl_2_ with air under ambient conditions by time‐resolved PXD over 60 h to assess possible hydrolysis/decomposition and the kinetics of any such processes in view of the outcomes if a tank and associated storage system were compromised (Figure 10). [Ni(NH_3_)_6_]Cl_2_ reacts over the duration of the experiment to yield a new phase, which can be indexed to a cell with approximate cell parameters of *a*≈7.90 Å, *b*≈7.80 Å, *c*≈3.80 Å, *α*=*β*=*γ*=90°. This is most likely a compound that contains both water and NH_3_ as ligands in a nickel‐centred complex because the hexaammine phase remains stable both under ammonia and an inert atmosphere. This hitherto unknown phase is only detectable after 5–10 h, and even after 60 h of exposure to air, the sample still contains detectable amounts of [Ni(NH_3_)_6_]Cl_2_. One could assume, therefore, that in the event of a hypothetical tank rupture under ambient conditions, any formation of ammonia would be slow and not considerable over hour timescales. Our further investigations of the formation and identity of the aqua‐ammine phase will be presented elsewhere.

**Figure 11:**
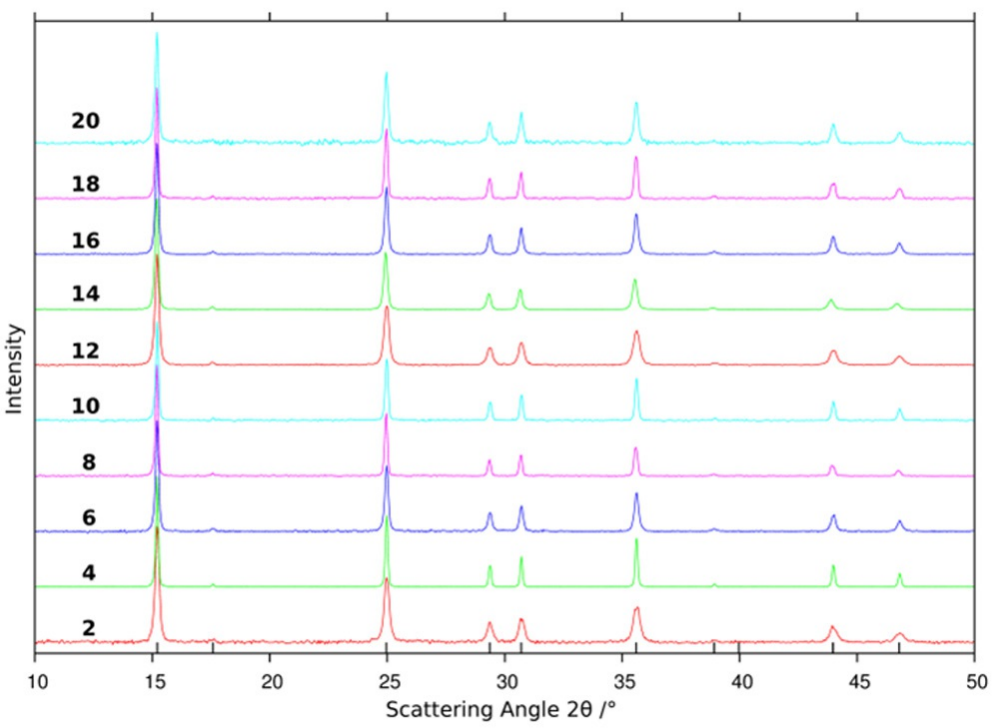
PXD patterns of [Ni(NH_3_)_6_]Cl_2_ after every second loading cycle between cycles 2–20 (bottom to top). The numbers next to the patterns signify the number of loading cycles. The tick marks on the x‐axis denote the calculated reflection positions for [Ni(NH_3_)_6_]Cl_2_.

One of the key performance parameters for any potential reversible gas storage material is its cyclability. Therefore, we performed cycling tests on the most promising of the nickel ammines, NiCl_2_. The uptake and release behaviour of the chloride was investigated over 20 cycles (20 uptake and 19 release steps). Neither the TG‐DTA curves (Figure S23 and Table S36) nor the PXD patterns (Figure 11) show any evidence of a significant loss of storage capacity or sample degradation. Indeed, the ammonia‐storage capacity fluctuates only between 99.0 and 97.2 % of the theoretical maximum over 20 cycles (to achieve 98.5 % of the maximum in the 20^th^ cycle). Throughout, besides ammonia, only trace amounts of nitrogen and hydrogen are detected in mass spectra of the gaseous products (Figure S24). Slight deviations in the decomposition temperature across cycles, as depicted in the TG‐DTA profiles, can likely be attributed to variations in the ammonia partial pressure from the use of small (relative to the sample holder) and differing sample masses (7–16 mg) during the TG‐DTA experiments.^42^ This was unavoidable as only small portions of the sample were taken for thermal analysis during cycling so as to maintain the integrity of the overall experiment. More comprehensive studies with regard to the kinetics and cycling properties of the ammines will be the subject of further study, but even after prolonged cycling and/or air exposure, the degradation products would most probably contain nickel hydroxides, nickel oxides and/or nickel metal. Nickel halides can be obtained easily and inexpensively from such compounds by treatment with the corresponding hydro‐halogen gas or acid.

## Conclusions

Nickel dihalide ammines are suitable materials for the reversible storage of significant amounts of NH_3_ (≈24–43 wt %). Our work shows that their thermal decomposition under relatively mild conditions combined with their easy production under ambient conditions place nickel ammine halides among the most promising materials for ammonia storage. Pure, stoichiometric hexaammine halides could be produced from the respective NiX_2_ (X=Cl, Br, I) halides by treatment with NH_3_ gas within 1 h at room temperature and atmospheric pressure. This reaction is strongly exothermic and leads to the formation of very small crystallites. The NiX_2_ systems can be cycled readily with ammonia. Contrary to some other ammonia‐storage materials, nickel halide hexaammines exhibit a relatively high stability in air as has been demonstrated for [Ni(NH_3_)_6_]Cl_2_. This characteristic is especially important in the case of a rupture of a potential ammonia store.

Thermal desorption can be resolved into three reaction steps, and NH_3_ is released upon heating. Our diffraction results demonstrate that only the respective diammines are readily observed in situ in each system. The diammine intermediate phases, Ni(NH_3_)_2_X_2_, adopt the Cd(NH_3_)_2_Cl_2_ structure type.

## Experimental Section

[Ni(H_2_O)_6_]Cl_2_ (99.9 %, Aldrich) was dehydrated by heating at 250 °C for 12 h in air before use. NiBr_2_ (99 %, Aldrich), NiI_2_ (synthesis grade, Aldrich) and NH_3_ (anhydrous, BOC) were used as received from the supplier. All the anhydrous nickel dihalides were characterised before use by using PXD.

The anhydrous nickel halides and the reaction products were handled in an Ar‐filled glovebox (Alpha/Omega series, Saffron Scientific Instruments, <10 ppm both H_2_O and O_2_) to prevent hydrate formation. Typically, the anhydrous nickel dihalide salt (500 mg–1 g) was sealed in a flask exposed to a flow of NH_3_ gas (≈50 mL min^−1^) at ambient pressure and RT for 1 h to obtain the respective hexaammines. The diammine samples for FTIR spectroscopy and SEM imaging were produced by heating samples of the hexaammine products (≈750 mg) in a vertical furnace in a glass tube flushed with N_2_ gas (flow rate of ≈50 mL min^−1^). The hexaammines were heated at 10 K min^−1^ and then held for 1 h at 393 K for the Cl system and 413 K for Br and I systems. Full decomposition to the diammines could be observed easily by a colour change to green (X=Cl, Br) or orange (X=I). For the cycling experiment, NiCl_2_ (≈1.25 g) was sealed in a flask and exposed alternately to NH_3(g)_ at RT for 1 h for uptake and heated at 5 K min^−1^ to 600 K for 2 h for release. A series of 20 subsequent cycles (20 uptake and 19 release steps) was performed. Small amounts of sample (≈70 mg) were taken from the hexaammine after every second cycle to perform thermal analysis and PXD experiments.

PXD patterns were collected for samples (≈10 mg, ≈10 μL) of the various products mounted in capillaries (0.7 mm diameter, Hilgenberg), which were prepared in a glovebox. Diffraction data were collected by using a Bruker D8 Advance powder diffractometer with CuK_*α*1_ radiation for the RT measurements. Data for phase identification were typically collected for 5°≤2 *θ*≤85° with a 0.05° step size and scan times of ≈20 min. Diffraction data were compared to patterns calculated from ICSD reference structures using POWDERCELL 2.4 and the CRYSCAL suite.^43^, ^44^ Additional data for the structure refinement of selected samples for X=Cl, I were collected over longer scan times (see refinement details in the Supporting Information). In situ variable‐temperature and ambient reactivity measurements were performed by using a PANalytical X′Pert Pro with CuK_*α*1_ radiation in Bragg–Brentano geometry. The RT refinement data for [Ni(NH_3_)_6_]Br_2_ were obtained by using the same geometry and an air‐sensitive sample holder with Mylar windows. For variable‐temperature experiments, an Anton Paar HK1200N high‐temperature cell with Kapton windows was used to heat the samples to the necessary temperatures under a constant flow of Ar gas (BOC, 99.998 %, ≈20 mL min^−1^). The samples were heated at 5 K min^−1^ to the respective target temperatures in steps of 20 K (300–500 K for X=Cl, 300–480 K for X=Br and 300–4440 K for X=I) and then held at the various measurement temperatures while data were collected. The patterns were recorded in the 2 *θ* range of 10–90° with a step size of 0.05° for 1.25 h. The data for the Rietveld refinement of the diammines were obtained from scans measured from 2 *θ*=10–85° at 373 K for X=Cl, Br and 393 K for X=I. The ambient reactivity experiment was performed with a flat plate‐mounted sample. Measurements from 2 *θ*=10–45° (step size of 0.05°) were performed sequentially with a scan duration of 30 min in each case over a cumulative period of 63 h. A structure‐less profile was fitted for refineable data using the Le Bail method within Jana2006.^26^ If possible, the structure was then solved using Superflip^25^ and the solutions compared to literature data to minimise the risk of employing an incorrect starting model. The data were subsequently treated by full‐matrix least‐squares refinement using Jana2006. Bond valence calculations were performed using the programme VALENCE^45^ with values of *R*_0_=1.308 and *b*=0.37.^46^

Thermogravimetric ammonia uptake measurements for NiCl_2_ were performed by using a Rubotherm IsoSORP magnetic suspension balance and gas dosing system. NiCl_2_ powder (251.4 mg) was contained in a glass sample holder within the reaction chamber. The sample was held under dynamic vacuum for 30 min before ammonia uptake to minimise the possibility of water uptake. The sample was exposed to a static atmosphere of ammonia gas at 1 bar, and the mass change of the sample was monitored.

TG‐DTA–MS experiments were conducted under flowing Ar (99.998 %, BOC) by using a Netzsch 409 PC STA instrument coupled to a Hiden Analytical HPR 20 mass spectrometer at an initial heating rate of 5 K min^−1^ and a heating rate of 4 K min^−1^ for cycling studies. Additional thermal measurements for the Kissinger analyses were performed with heating rates of 2, 10, 15 and 20 K min^−1^. [Ni(NH_3_)_6_]Cl_2_ (15±1 mg) was used in each case in these variable heating rate experiments. The STA instrument was located in an Ar‐filled glovebox (O_2_, H_2_O<3 ppm, UNILab, MBraun). This configuration enables even samples of extreme air sensitivity to be analysed without contact with air. TG‐DTA–MS samples (≈25 mg) were loaded in cylindrical Al_2_O_3_ sample holders.

Combustion analyses were conducted on samples (≈2 mg) by using an Exeter Analytical CE‐440 elemental analyzer to obtain values for hydrogen content. Contact with air during the sample preparation was minimised (<1 min) to avoid sample degradation.

FTIR spectroscopy was conducted by using a Shimadzu FTIR‐8400S spectrometer by accumulating 24 spectra at a resolution of 2 cm^−1^ in the range of 4000–600 cm^−1^. The solid‐state samples (≈10 mg) were measured by attenuated total reflectance (ATR) by pressing the samples on to the diamond crystal of a MIRacle (PIKE) sample holder. The inevitable air exposure time of the sample transfer to the FTIR spectrometer was minimised to ≈5 s.

Images of the diammines were obtained by using a XL 30 ESEM (Philips) SEM with a tungsten filament using a 15 kV acceleration voltage. The EDX spectra were recorded by using a 10 mm^2^ silicon drifted EDX detector (X‐act, Oxford Instruments) and calibrated with Cu. High‐magnification images of the hexaammines were taken by using a ΣIGMA field‐emission SEM (Carl Zeiss). An acceleration voltage of 15 kV was applied using a target‐to‐sample distance of 8.5 mm. Samples were coated with gold before the measurement. Unavoidable air contact upon transfer was minimised to less than 1 min to preserve the sample from degradation.

## Supporting information

As a service to our authors and readers, this journal provides supporting information supplied by the authors. Such materials are peer reviewed and may be re‐organized for online delivery, but are not copy‐edited or typeset. Technical support issues arising from supporting information (other than missing files) should be addressed to the authors.

Supplementary
