# Supplementary material for: Facile Uptake and Release of Ammonia by Nickel Halide Ammines
Source: ChemSusChem. 2016 May 3;9(11):1312–21. doi: 10.1002/cssc.201600140 (PMC5084821; doi:10.1002/cssc.201600140)
Supplement: Supplementary file 1 — Supplementary [file CSSC-9-1312-s001.pdf]

CHEMISTRY & SUSTAINABILITY
CHEMSUSCHEM
ENERGY & MATERIALS

Supporting Information

Facile Uptake and Release of Ammonia by Nickel Halide Ammines

Joachim Breternitz,[a] Yury E. Vilk,[a, b] Elsa Giraud,[a, c] Hazel Reardon,[a] Tuan K. A. Hoang,[a] Agata Godula-Jopek,[d, e] and Duncan H. Gregory*[a]

cssc_201600140_sm_miscellaneous_information.pdf

1) Ammonia uptake

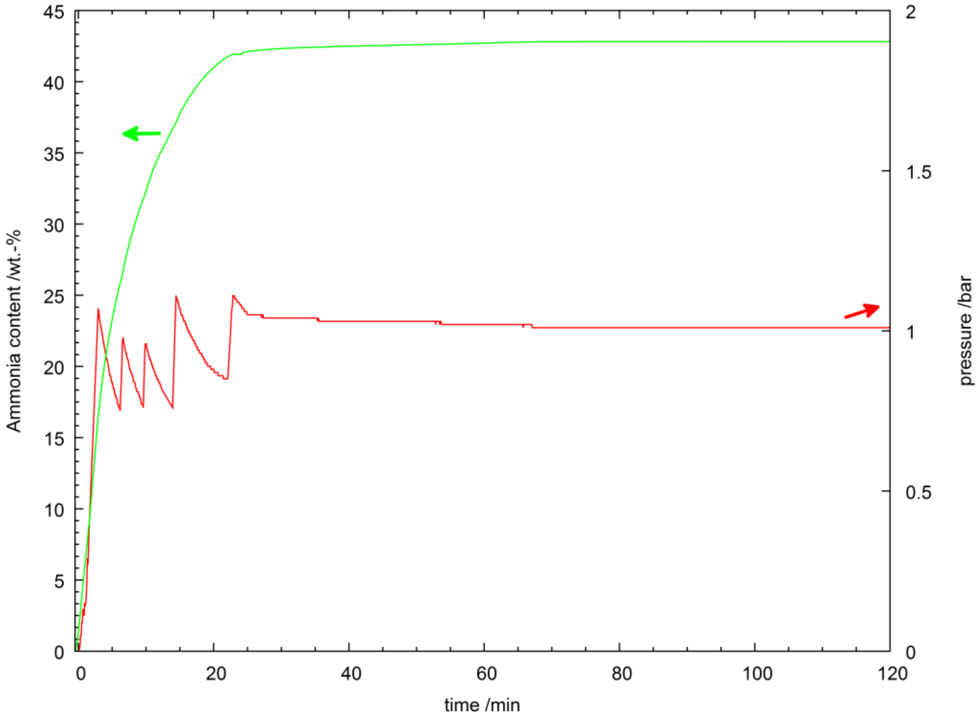

Figure S1: Kinetic gravimetric uptake measurement performed on $NiCl_2$ at room temperature and 1 bar NH_3.

2) FTIR

Table S1: Maxima and relative intensities of the IR bands for $[Ni(NH_3)_6]X_2$ compared with the literature values

	$[Ni(NH_3)_6]Cl_2$[S1]	$[Ni(NH_3)_6]Cl_2$	$[Ni(NH_3)_6]Br_2$	$[Ni(NH_3)_6]I_2$
$\nu_a(NH_3)$	3345 (s)	3342.75 (s)	3337.93 (m)	3331.18 (m)
$\nu_s(NH_3)$	3190 (w)	3192.59 (sh)	3177.41 (sh)	3172.89 (sh)
$\delta_a(HNH)$	1605 (m)	1604.83 (m)	1598.08 (m)	1595.18 (m)
$\delta_s(HNH)$	1186 (vs)	1166.97 (s)	1176.62 (s)	1197.83 (s)
$\rho(NH_3)$	684 (s)	673.18 (vs)	660.64 (vs)	648.10 (vs)

vs: very strong, s: strong, m: medium, sh: shoulder

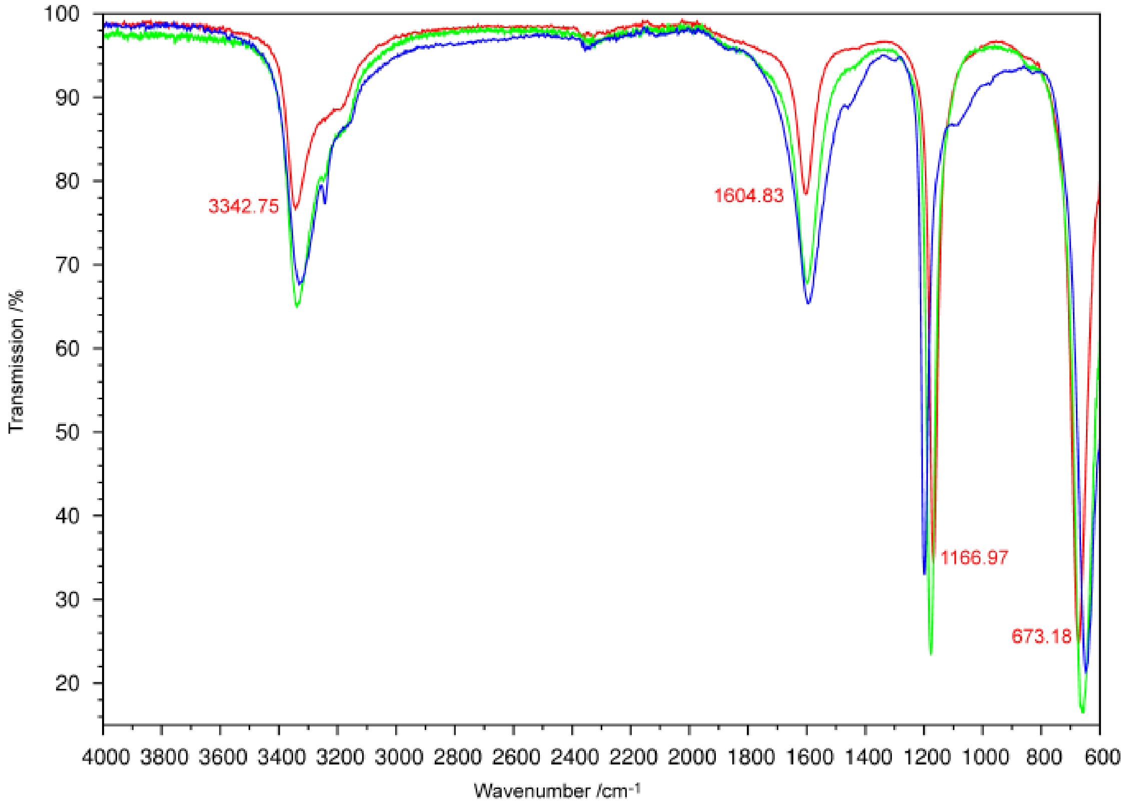

Figure S2: ATR-FTIR spectra for $[Ni(NH_3)_6]X_2$ where X = Cl (red), Br (green) and I (blue). The wavenumbers of the absorption maxima given in the figure are those for $[Ni(NH_3)_6]Cl_2$.

Table S2: Maxima and relative intensities of the IR bands for $Ni(NH_3)_2X_2$ compared with the literature values

	$Ni(NH_3)_2Cl_2$ [S2]	$Ni(NH_3)_2Cl_2$	$Ni(NH_3)_2Br_2$	$Ni(NH_3)_2I_2$
$\nu_a(NH_3)$	3347	3345.86 (m)	3335.86 (m)	3323.00 (m)
$\nu_s(NH_3)$	3264	3265.11 (m)	3254.39 (m)	3239.39 (m)
$2\delta_a$(HNH)	3171	3167.21 (w)	3160.78 (w)	3146.49 (w)
δ_a(HNH)	1611	1607.30 (m)	1604.07 (m)	1596.21 (m)
δ_s(HNH)	1405	1405.83 (w)	1411.37 (w)	1395.20 (m)
δ_s(HNH)	1282	1281.06 (w)	1270.43/1256.57 (w)	not observed
δ_s(HNH)	1246	1239.01 (vs)	1243.63 (vs)	1239.94 (vs)
$\rho(NH_3)$	675	674.62 (m)	672.66 (m)	659.92 (sh)

vs: very strong, s: strong, m: medium, sh: shoulder, w: weak

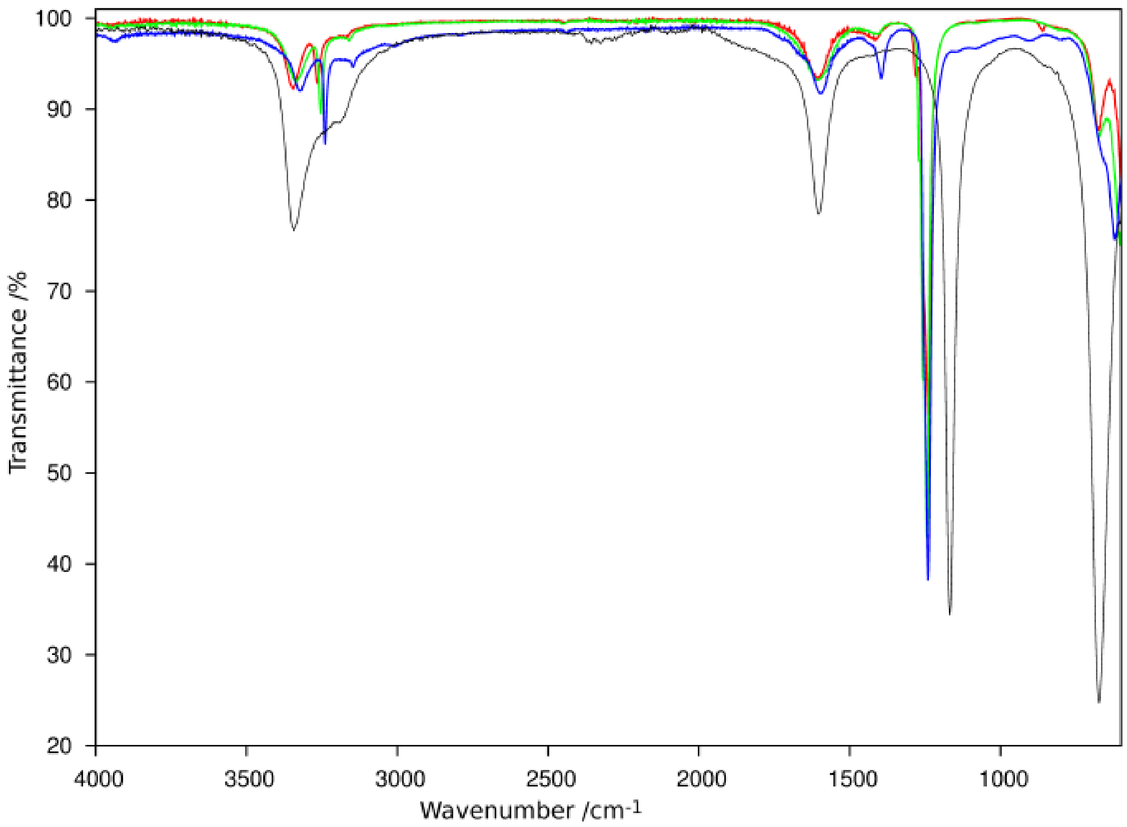

Figure S3: ATR-FTIR spectra for NiX_2·$(NH_3)_2$ where X = Cl (red), Br (green) and I (blue) compared to the spectrum for $[Ni(NH_3)_6]Cl_2$ (black). The wavenumbers of the absorption maxima given in the figure are those for $[Ni(NH_3)_6]Cl_2$.

3) STA analyses

Table S3 Thermal decomposition (STA) results for $[Ni(NH_3)_6]X_2$ (X = Cl, Br, I) with a heating ramp of 5 K·min^{-1}

**Sample**	$N(NH_3)$	Mass changes obs.(calc.) / wt.-%		TG (STA) Temperatures / K		Peak Temperatures / K	
	final	Total	Stepwise	Onset	Final	DTA	MS
**$[Ni(NH_3)_6]Cl_2$**	2	28.2 (29.4)	28.2 (29.4)	400	456	448	485
	0	42.8 (44.0)	14.6 (14.6)	547	580	575	587
**$[Ni(NH_3)_6]Br_2$**	2	21.1 (21.2)	21.1 (21.2)	416	475	468	466
	0	31.3 (31.7)	10.2 (10.5)	532	579	575	588
**$[Ni(NH_3)_6]I_2$**	2	15.3 (16.4)	15.3 (16.4)	427	482	477	494
	0	23.4 (24.6)	8.1 (8.2)	514	549	543	555

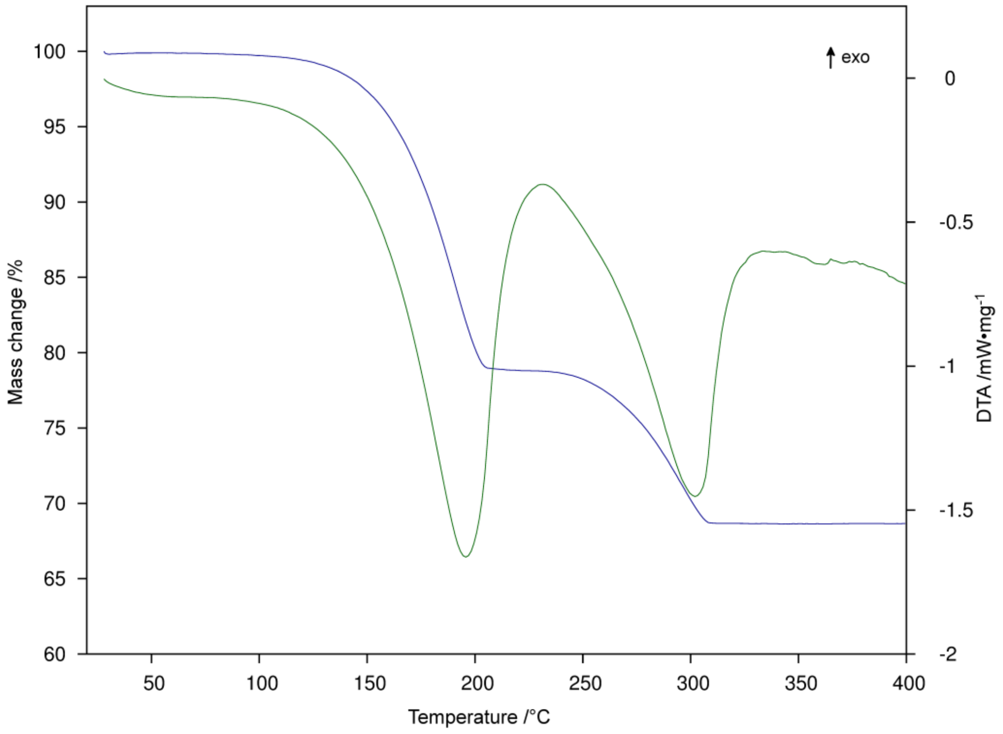

Figure S4: STA profile for $[Ni(NH_3)_6]Br_2$ at a heating rate of 5 K min^{-1} with TG (blue) and DTA (green).

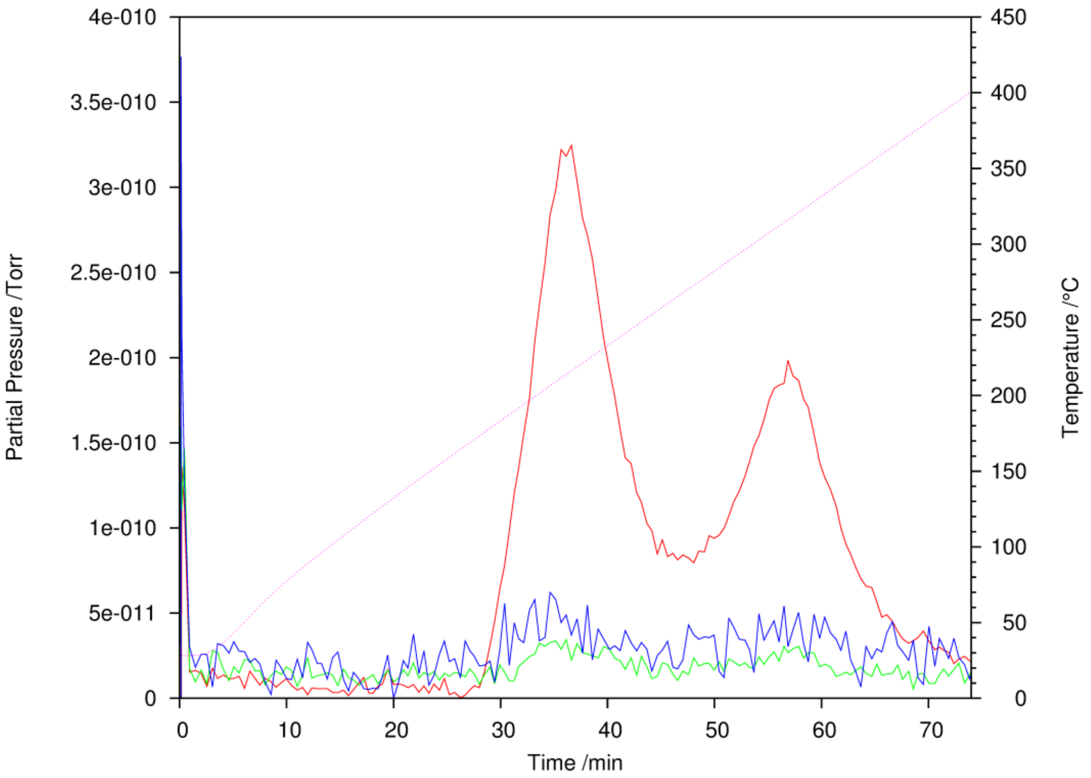

Figure S5: MS curves for the gas evolved from the STA with m/z = 2(H_2^+, blue), 17(NH_3^+, red) and 28(N_2^+, green) for $[Ni(NH_3)_6]Br_2$ (figure S3). The STA temperature profile is denoted by magenta dots.

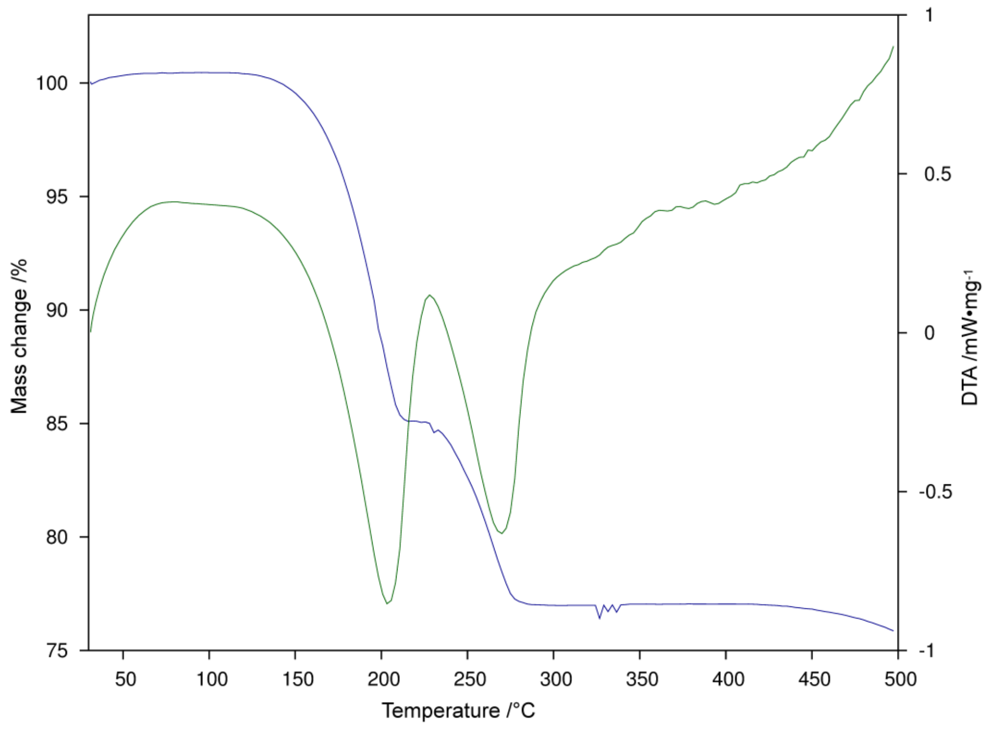

Figure S6: STA profile for $[Ni(NH_3)_6]I_2$ at a heating rate of 5 K min^{-1} with TG (blue) and DTA (green).

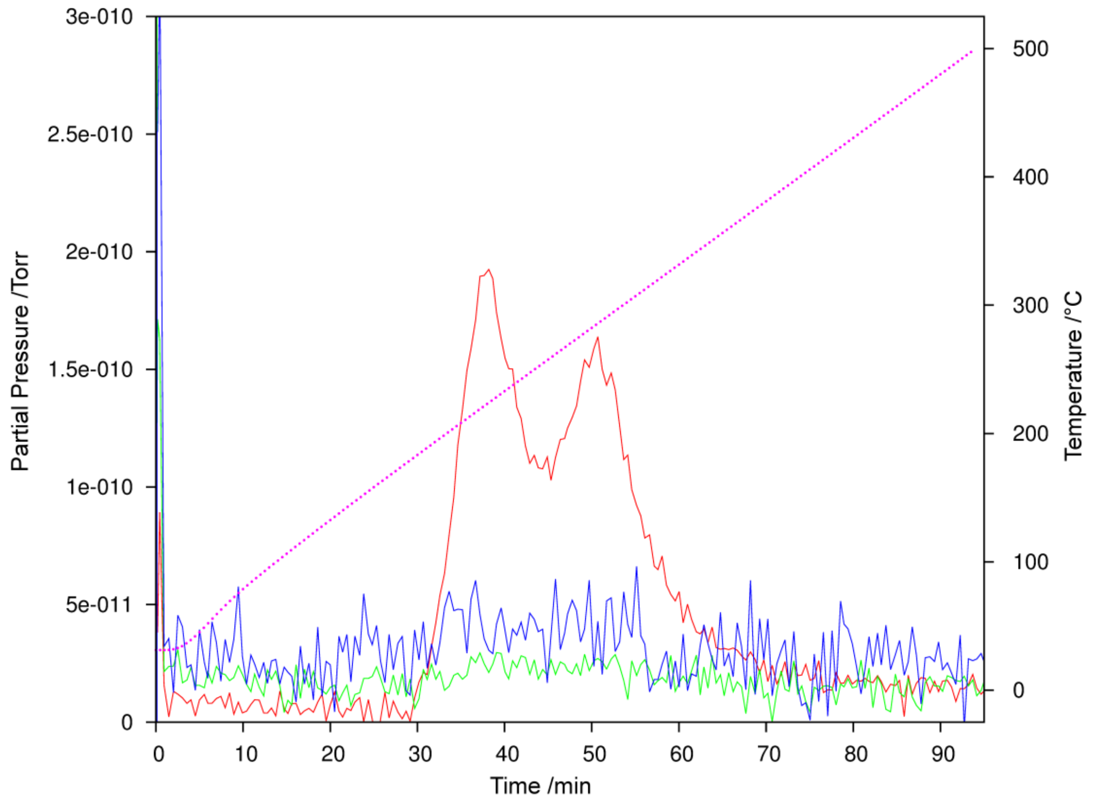

Figure S7: MS curves for the outstream gas from the STA with m/z = 2(H_2^+, blue), 17(NH_3^+, red) and 28(N_2^+, green) for $[Ni(NH_3)_6]I_2$ (figure S5). The STA temperature profile is denoted by magenta dots.

4) SEM Images

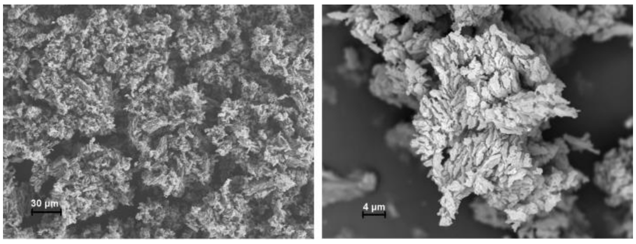

Figure S8: SEM images of $[Ni(NH_3)_6]Cl_2$ samples produced by ammoniation at room temperature.

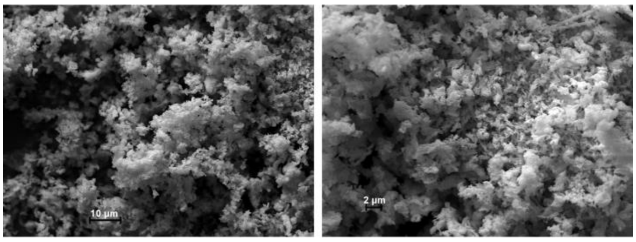

Figure S9: SEM images of $[Ni(NH_3)_6]Br_2$ samples produced by ammoniation at room temperature.

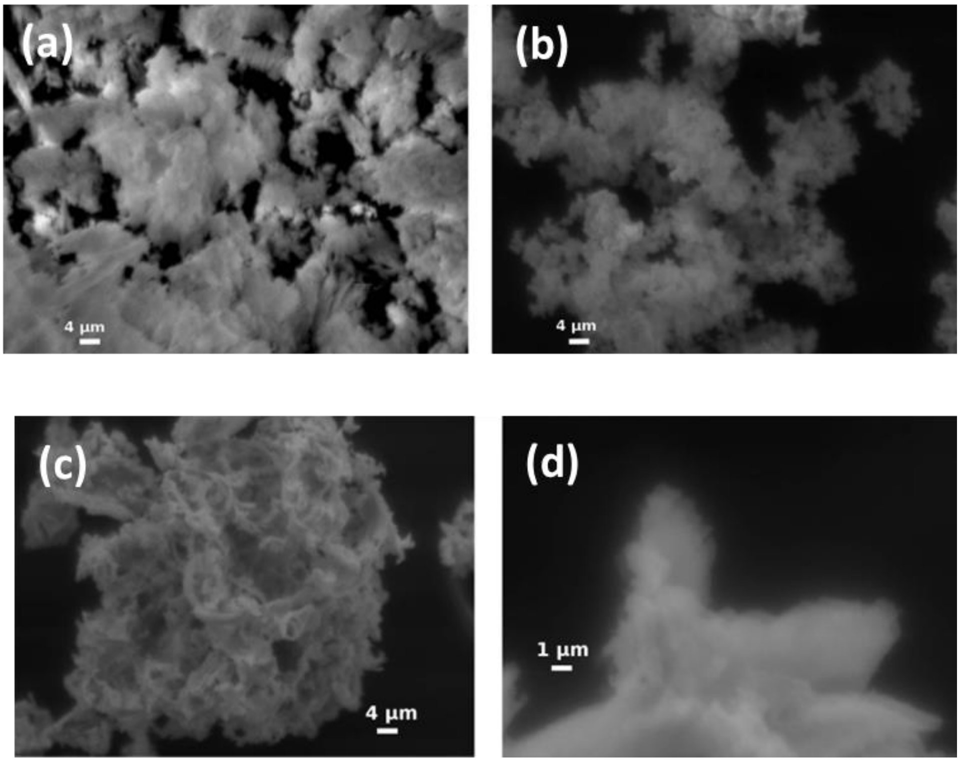

Figure S10: SEM images of (a) $Ni(NH_3)_2Cl_2$, (b) $Ni(NH_3)_2Br_2$ and (c,d) $Ni(NH_3)_2I_2$.

5) Crystallographic Data for $[Ni(NH_3)_6]Cl_2$

Table S4: Crystal data

$H_{18}N_6Ni\cdot 2(Cl)$	$F(000) = 488$
$M_r = 231.8$	$D_x = 1.506$ Mg m^{-3}
Cubic, $Fm\bar{3}m$	Cu $K\alpha_1$ radiation, $\lambda = 1.54056$ Å
Hall symbol: -F 4 2 3	$T = 293$ K
$a = 10.0744$ (3) Å	Particle morphology: irregular powder, visual examination
$V = 1022.49$ (6) Å^3	pale violet
$Z = 4$	cylinder, 0.5 × 5 mm

Table S5: Data collection parameters

Bruker d8 diffractometer	Scan method: continuous
Radiation source: sealed X-ray tube, Bruker d8	$2\theta_{min} = 5°$, $2\theta_{max} = 107.98°$, $2\theta_{step} = 0.03°$
Ge 111 monochromator	

Table S6: Refinement parameters

$R_p = 0.036$	0 restraints
$R_{wp} = 0.046$	0 constraints
$R_{exp} = 0.032$	H-atom parameters constrained
$R(F) = 0.033$	Weighting scheme based on measured s.u.'s
$\chi^2 = 2.074$	$(\Delta/\sigma)_{max} = 0.006$
4120 data points	Background function: 12 Legendre polynoms
Profile function: Pseudo-Voigt	Preferred orientation correction: none
25 parameters	

Table S7: Fractional atomic coordinates and isotropic or equivalent isotropic displacement parameters /Å^2. Asterisks signify isotropically refined thermal displacement parameters (U_{iso}), otherwise U_{eq} signifies the equivalent parameter in the anisotropically refined case.

	x	*Y*	*z*	U_{iso}*/U_{eq}	Occ. (<1)
Ni1	0	0	0	0.029 (3)*	
Cl1	0.25	0.25	0.25	0.040 (3)*	
N1	0.2139 (8)	0	0	0.052 (5)	
H1n1	0.242647	0.076382	0.02819	0.0624*	0.125
H2n1	0.242647	-0.062605	0.052054	0.0624*	0.125
H3n1	0.242647	-0.013778	-0.080244	0.0624*	0.125

Table S8: Atomic displacement parameters /Å^2

	U^{11}	U^{22}	U^{33}	U^{12}	U^{13}	U^{23}
N1	0.032 (9)	0.062 (7)	0.062 (7)	0	0	0

Table S9: Selected geometric parameters /Å, º

N1—H1n1	0.87	N1—H3n1^{iii}	0.87
N1—H1n1^{i}	0.87	N1—H3n1^{iv}	0.87
N1—H1n1^{ii}	0.87	N1—H3n1^{v}	0.87
N1—H1n1^{iii}	0.87	N1—H3n1^{vi}	0.87
N1—H1n1^{iv}	0.87	N1—H3n1^{vii}	0.87
N1—H1n1^{v}	0.87	H1n1—H1n1^{iv}	0.568
N1—H1n1^{vi}	0.87	H1n1—H1n1^{vii}	0.6866
N1—H1n1^{vii}	0.87	H1n1—H2n1^{i}	0.8202
N1—H2n1	0.87	H1n1—H2n1^{ii}	0.4246
N1—H2n1^{i}	0.87	H1n1—H2n1^{v}	0.2776
N1—H2n1^{ii}	0.87	H1n1—H3n1^{i}	0.8202
N1—H2n1^{iii}	0.87	H1n1—H3n1^{iii}	0.4246
N1—H2n1^{iv}	0.87	H1n1—H3n1^{vi}	0.1503
N1—H2n1^{v}	0.87	H2n1—H2n1^{vi}	0.1503
N1—H2n1^{vi}	0.87	H2n1—H3n1^{i}	0.8202
N1—H2n1^{vii}	0.87	H2n1—H3n1^{ii}	0.4246
N1—H3n1	0.87	H2n1—H3n1^{iv}	0.568
N1—H3n1^{i}	0.87	H2n1—H3n1^{vii}	0.6866
N1—H3n1^{ii}	0.87	H3n1—H3n1^{v}	0.2776
H1n1—N1—H1n1^{i}	141.06	H2n1^{ii}—N1—H3n1^{ii}	109.47
H1n1—N1—H1n1^{ii}	83.62	H2n1^{ii}—N1—H3n1^{iii}	56.25
H1n1—N1—H1n1^{iii}	83.62	H2n1^{ii}—N1—H3n1^{iv}	46.48
H1n1—N1—H1n1^{iv}	38.11	H2n1^{ii}—N1—H3n1^{v}	117.8
H1n1—N1—H1n1^{v}	124.38	H2n1^{ii}—N1—H3n1^{vi}	38.11
H1n1—N1—H1n1^{vi}	117.8	H2n1^{ii}—N1—H3n1^{vii}	124.38
H1n1—N1—H1n1^{vii}	46.48	H2n1^{iii}—N1—H2n1^{iv}	9.91
H1n1—N1—H2n1	109.47	H2n1^{iii}—N1—H2n1^{v}	139.72
H1n1—N1—H2n1^{i}	56.25	H2n1^{iii}—N1—H2n1^{vi}	92.93
H1n1—N1—H2n1^{ii}	28.25	H2n1^{iii}—N1—H2n1^{vii}	74.14
H1n1—N1—H2n1^{iii}	131.2	H2n1^{iii}—N1—H3n1	28.25
H1n1—N1—H2n1^{iv}	136.62	H2n1^{iii}—N1—H3n1^{i}	131.2
H1n1—N1—H2n1^{v}	18.36	H2n1^{iii}—N1—H3n1^{ii}	56.25
H1n1—N1—H2n1^{vi}	100.68	H2n1^{iii}—N1—H3n1^{iii}	109.47
H1n1—N1—H2n1^{vii}	65.95	H2n1^{iii}—N1—H3n1^{iv}	117.8
H1n1—N1—H3n1	109.47	H2n1^{iii}—N1—H3n1^{v}	46.48
H1n1—N1—H3n1^{i}	56.25	H2n1^{iii}—N1—H3n1^{vi}	124.38
H1n1—N1—H3n1^{ii}	131.2	H2n1^{iii}—N1—H3n1^{vii}	38.11
H1n1—N1—H3n1^{iii}	28.25	H2n1^{iv}—N1—H2n1^{v}	141.06

H1n1—N1—H3n1^{iv}	74.14	H2n1^{iv}—N1—H2n1^{vi}	83.62
H1n1—N1—H3n1^{v}	92.93	H2n1^{iv}—N1—H2n1^{vii}	83.62
H1n1—N1—H3n1^{vi}	9.91	H2n1^{iv}—N1—H3n1	38.11
H1n1—N1—H3n1^{vii}	139.72	H2n1^{iv}—N1—H3n1^{i}	124.38
H1n1^{i}—N1—H1n1^{ii}	83.62	H2n1^{iv}—N1—H3n1^{ii}	46.48

Symmetry codes: (i) *x*, -*y*, -*z*; (ii) *x*, *z*, -*y*; (iii) *x*, -*z*, *y*; (iv) *x*, *y*, -*z*; (v) *x*, -*y*, *z*; (vi) *x*, -*z*, -*y*; (vii) *x*, *z*, *y*.

6) Crystallographic Data for $[Ni(NH_3)_6]Br_2$

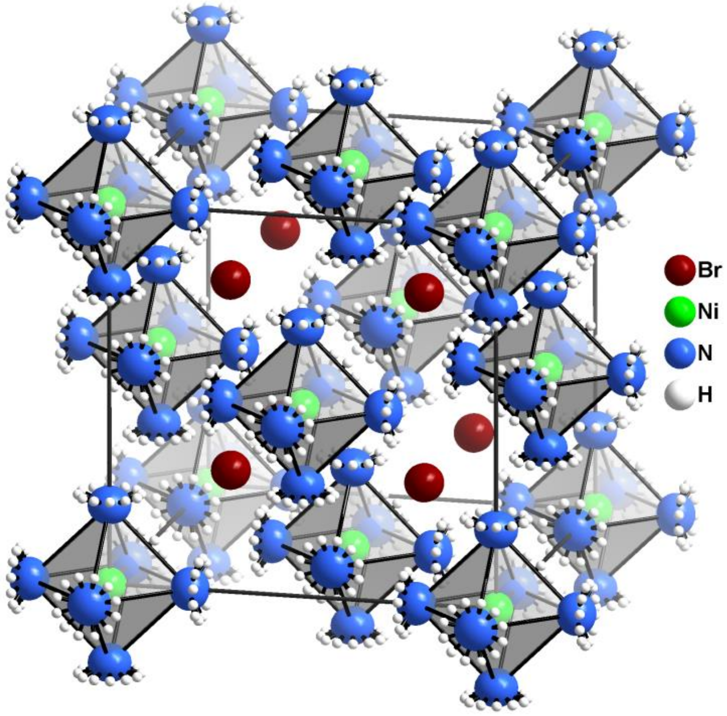

Figure S11: Representation of the structure of $[Ni(NH_3)_6]Br_2$. The atom ellipsoids (apart from H) represent 90 % spatial probability. The hydrogen atoms are represented as generic spheres.

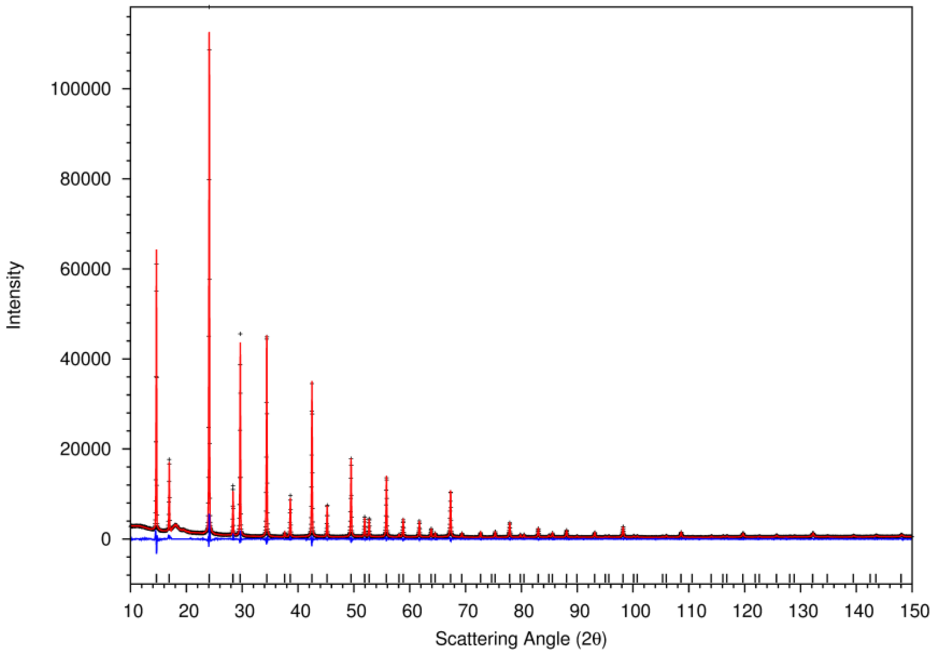

Figure S12: Rietveld refinement profile for $[Ni(NH_3)_6]Br_2$. The red line represents the calculated pattern, the black crosses the measured pattern with the difference represented by the blue line. Reflection positions are represented by black ticks.

Table S10: Crystal data

$H_{18}N_6Ni\cdot 2(Br)$	$F(000) = 632$
$M_r = 320.7$	$D_x = 1.903$ Mg m^{-3}
Cubic, $Fm\bar{3}m$	Cu $K\alpha_1$ radiation, $\lambda = 1.54056$ Å
Hall symbol: -F 4 2 3	$T = 293$ K
$a = 10.3826$ (3) Å	Particle morphology: irregular powder, visual examination
$V = 1119.22$ (6) Å^3	pale violet
$Z = 4$	flat sample, 5 x 5 mm

Table S11: Data collection parameters

PANalytical XPert Pro diffractometer	Scan method: continuous
Radiation source: sealed X-ray tube, PANalytical XPert Pro	$2\theta_{min} = 10.03°$, $2\theta_{max} = 149.95°$, $2\theta_{step} = 0.03°$
Ge 111 monochromator	

Table S12: Refinement parameters

R_p = 0.041	27 parameters
R_{wp} = 0.054	0 restraints
R_{exp} = 0.030	1 constraint
$R(F)$ = 0.019	H-atom parameters constrained
χ^2 = 3.240	Weighting scheme based on measured s.u.'s
4241 data points	$(\Delta/\sigma)_{max}$ = 0.030
Excluded region(s): from 17.303 to 20.557	Background function: Manual background combined with 10 Legendre polynoms
Profile function: Pseudo-Voigt	Preferred orientation correction: none

Table S13: Fractional atomic coordinates and isotropic or equivalent isotropic displacement parameters /Å^2. Asterisks signify isotropically refined thermal displacement parameters (U_{iso}), otherwise U_{eq} signifies the equivalent parameter in the anisotropically refined case.

	x	*y*	*z*	U_{iso}*/U_{eq}	Occ. (<1)
Br1	0.25	0.25	0.25	0.0435 (6)*	
Ni1	0	0	0	0.0312 (9)*	
N1	0.2073 (4)	0	0	0.050 (3)	
H1n1	0.235268	-0.055863	0.055863	0.0595*	0.25
H2n1	0.235268	-0.020447	-0.07631	0.0595*	0.25

Table S14: Atomic displacement parameters /Å^2

	U^{11}	U^{22}	U^{33}	U^{12}	U^{13}	U^{23}
N1	0.043 (5)	0.053 (4)	0.053 (4)	0	0	0

Table S15: Selected Geometric parameters /Å, º

N1—H1n1	0.87	N1—H2n1^{v}	0.87
N1—H1n1^{i}	0.87	N1—H2n1^{vi}	0.87
N1—H1n1^{ii}	0.87	N1—H2n1^{vii}	0.87
N1—H1n1^{iii}	0.87	H1n1—H2n1^{i}	0.8202
N1—H2n1	0.87	H1n1—H2n1^{ii}	0.4246
N1—H2n1^{i}	0.87	H1n1—H2n1^{iv}	0.4246
N1—H2n1^{ii}	0.87	H1n1—H2n1^{vii}	0.8202
N1—H2n1^{iii}	0.87	H2n1—H2n1^{v}	0.4246
N1—H2n1^{iv}	0.87	H2n1—H2n1^{vii}	0.8202
H1n1—N1—H1n1^{i}	141.06	H2n1—N1—H2n1^{vi}	109.47
H1n1—N1—H1n1^{ii}	83.62	H2n1—N1—H2n1^{vii}	56.25
H1n1—N1—H1n1^{iii}	83.62	H2n1^{i}—N1—H2n1^{ii}	83.62
H1n1—N1—H2n1	109.47	H2n1^{i}—N1—H2n1^{iii}	83.62
H1n1—N1—H2n1^{i}	56.25	H2n1^{i}—N1—H2n1^{iv}	28.25
H1n1—N1—H2n1^{ii}	28.25	H2n1^{i}—N1—H2n1^{v}	131.2
H1n1—N1—H2n1^{iii}	131.2	H2n1^{i}—N1—H2n1^{vi}	56.25
H1n1—N1—H2n1^{iv}	28.25	H2n1^{i}—N1—H2n1^{vii}	109.47
H1n1—N1—H2n1^{v}	131.2	H2n1^{ii}—N1—H2n1^{iii}	141.06
H1n1—N1—H2n1^{vi}	109.47	H2n1^{ii}—N1—H2n1^{iv}	56.25
H1n1—N1—H2n1^{vii}	56.25	H2n1^{ii}—N1—H2n1^{v}	109.47
H1n1^{i}—N1—H1n1^{ii}	83.62	H2n1^{ii}—N1—H2n1^{vi}	131.2
H1n1^{i}—N1—H1n1^{iii}	83.62	H2n1^{ii}—N1—H2n1^{vii}	28.25
H1n1^{i}—N1—H2n1	56.25	H2n1^{iii}—N1—H2n1^{iv}	109.47
H1n1^{i}—N1—H2n1^{i}	109.47	H2n1^{iii}—N1—H2n1^{v}	56.25
H1n1^{i}—N1—H2n1^{ii}	131.2	H2n1^{iii}—N1—H2n1^{vi}	28.25
H1n1^{i}—N1—H2n1^{iii}	28.25	H2n1^{iii}—N1—H2n1^{vii}	131.2
H1n1^{i}—N1—H2n1^{iv}	131.2	H2n1^{iv}—N1—H2n1^{v}	141.06
H1n1^{i}—N1—H2n1^{v}	28.25	H2n1^{iv}—N1—H2n1^{vi}	83.62
H1n1^{i}—N1—H2n1^{vi}	56.25	H2n1^{iv}—N1—H2n1^{vii}	83.62
H1n1^{i}—N1—H2n1^{vii}	109.47	H2n1^{v}—N1—H2n1^{vi}	83.62
H1n1^{ii}—N1—H1n1^{iii}	141.06	H2n1^{v}—N1—H2n1^{vii}	83.62
H1n1^{ii}—N1—H2n1	131.2	H2n1^{vi}—N1—H2n1^{vii}	141.06
H1n1^{ii}—N1—H2n1^{i}	28.25	N1—H1n1—H2n1^{i}	61.87
H1n1^{ii}—N1—H2n1^{ii}	109.47	N1—H1n1—H2n1^{ii}	75.88
H1n1^{ii}—N1—H2n1^{iii}	56.25	N1—H1n1—H2n1^{iv}	75.88
H1n1^{ii}—N1—H2n1^{iv}	56.25	N1—H1n1—H2n1^{vii}	61.87
H1n1^{ii}—N1—H2n1^{v}	109.47	H2n1^{i}—H1n1—H2n1^{ii}	135

Symmetry codes: (i) *x*, -*y*, -*z*; (ii) *x*, *z*, -*y*; (iii) *x*, -*z*, *y*; (iv) *x*, *y*, -*z*; (v) *x*, -*y*, *z*; (vi) *x*, -*z*, -*y*; (vii) *x*, *z*, *y*.

7) Crystallographic Data for $[Ni(NH_3)_6]I_2$

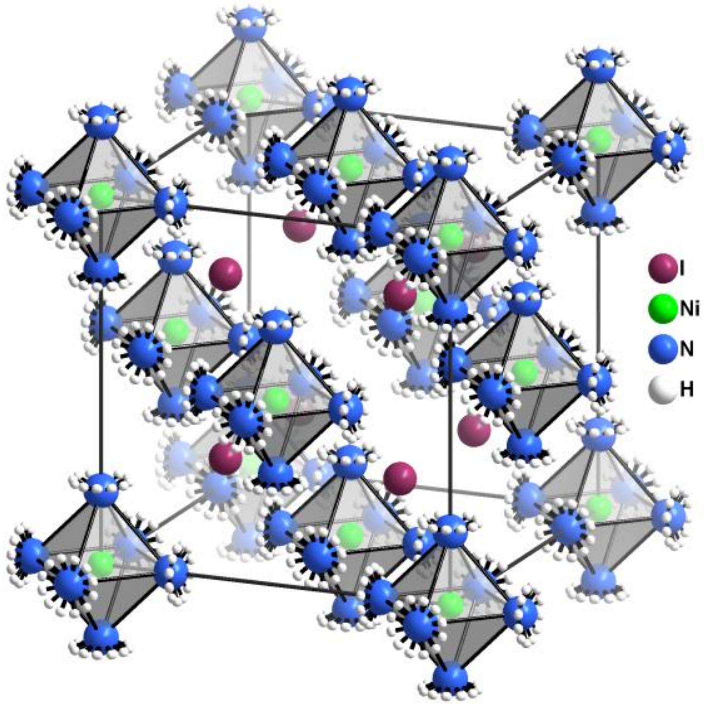

Figure S13: Representation of the structure of $[Ni(NH_3)_6]I_2$. The atom ellipsoids (apart from H) represent 90 % spatial probability. The hydrogen atoms are represented as generic spheres.

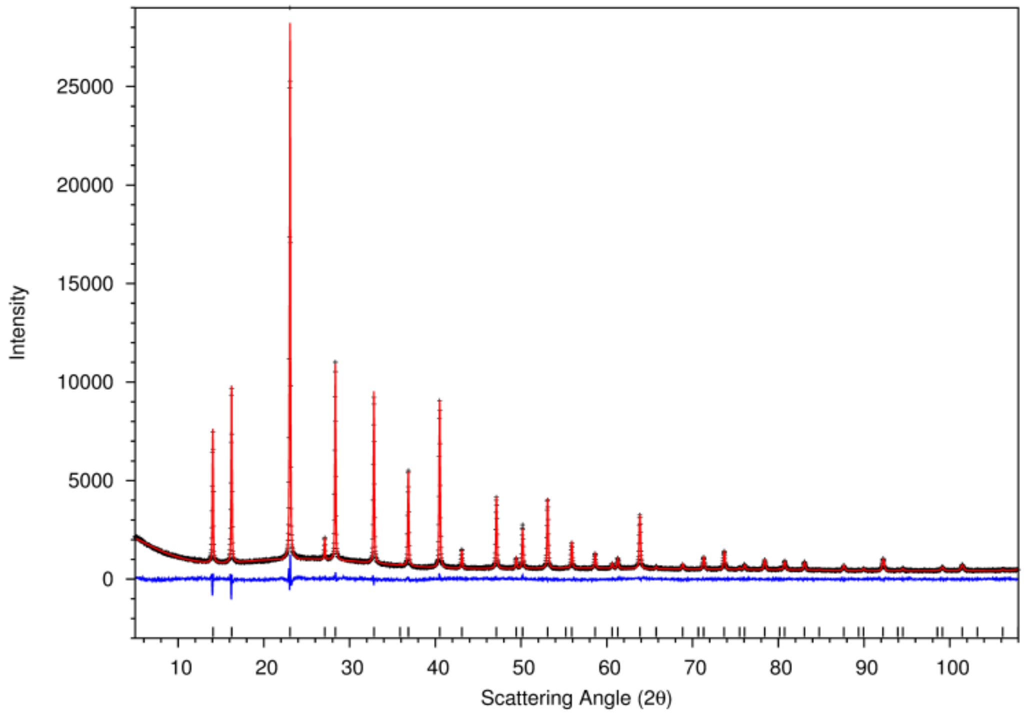

Figure S14: Rietveld refinement profile for $[Ni(NH_3)_6]I_2$. The red line represents the calculated pattern, the black crosses the measured pattern with the difference represented by the blue line. Reflection positions are represented by black ticks.

Table S16: Crystal data

$H_{18}N_6Ni\cdot 2(I)$	$F(000) = 776$
$M_r = 414.7$	$D_x = 2.127$ Mg m^{-3}
Cubic, $Fm\bar{3}m$	Cu $K\alpha_1$ radiation, $\lambda = 1.54056$ Å
Hall symbol: -F 4 2 3	$T = 293$ K
$a = 10.89991$ (15) Å	Particle morphology: irregular powder, visual examination
$V = 1295.00$ (3) Å^3	white
$Z = 4$	cylinder, 0.5 × 5 mm

Table S17: Data collection parameters

Bruker d8 diffractometer	Scan method: continuous
Radiation source: sealed X-ray tube, Bruker d8	$2\theta_{min} = 5°$, $2\theta_{max} = 107.98°$, $2\theta_{step} = 0.03°$
Ge 111 monochromator	

Table S18: Refinement parameters

$R_p = 0.034$	0 restraints
$R_{wp} = 0.044$	1 constraint
$R_{exp} = 0.035$	H-atom parameters constrained
$R(F) = 0.040$	Weighting scheme based on measured s.u.'s
$\chi^2 = 1.588$	$(\Delta/\sigma)_{max} = 0.041$
4120 data points	Background function: 12 Legendre polynoms
Profile function: Pseudo-Voigt	Preferred orientation correction: none
24 parameters	

Table S19: Fractional atomic coordinates and isotropic or equivalent isotropic displacement parameters /Å^2. Asterisks signify isotropically refined thermal displacement parameters (U_{iso}), otherwise U_{eq} signifies the equivalent parameter in the anisotropically refined case.

	x	y	z	U_{iso}*/U_{eq}	Occ. (<1)
I1	0.25	0.25	0.25	0.0376 (10)*	
Ni1	0	0	0	0.024 (2)*	
N1	0.1993 (8)	0	0	0.042 (5)*	
H1n1	0.225934	-0.054656	0.051727	0.0499*	0.125
H2n1	0.225934	-0.017469	-0.073197	0.0499*	0.125
H3n1	0.225934	0.072125	0.02147	0.0499*	0.125

Table S20: Selected Geometric parameters /Å, º

N1—H1n1	0.87	N1—H3n1^{iv}	0.87
N1—H1n1^{i}	0.87	N1—H3n1^{v}	0.87
N1—H1n1^{ii}	0.87	N1—H3n1^{vi}	0.87
N1—H1n1^{iii}	0.87	N1—H3n1^{vii}	0.87
N1—H1n1^{iv}	0.87	H1n1—H1n1^{vi}	0.0451
N1—H1n1^{v}	0.87	H1n1—H2n1^{i}	0.8202
N1—H1n1^{vi}	0.87	H1n1—H2n1^{ii}	0.4246
N1—H1n1^{vii}	0.87	H1n1—H2n1^{iv}	0.468
N1—H2n1	0.87	H1n1—H2n1^{vii}	0.7808
N1—H2n1^{i}	0.87	H1n1—H3n1^{i}	0.8202
N1—H2n1^{ii}	0.87	H1n1—H3n1^{iii}	0.4246
N1—H2n1^{iii}	0.87	H1n1—H3n1^{v}	0.3808
N1—H2n1^{iv}	0.87	H1n1—H3n1^{vii}	0.859
N1—H2n1^{v}	0.87	H2n1—H2n1^{v}	0.3808
N1—H2n1^{vi}	0.87	H2n1—H2n1^{vii}	0.859
N1—H2n1^{vii}	0.87	H2n1—H3n1^{i}	0.8202
N1—H3n1	0.87	H2n1—H3n1^{ii}	0.4246
N1—H3n1^{i}	0.87	H2n1—H3n1^{vi}	0.0451
N1—H3n1^{ii}	0.87	H3n1—H3n1^{iv}	0.468
N1—H3n1^{iii}	0.87	H3n1—H3n1^{vii}	0.7808
H1n1—N1—H1n1^{i}	141.06	H2n1^{iii}—N1—H3n1^{iv}	2.97
H1n1—N1—H1n1^{ii}	83.62	H2n1^{iii}—N1—H3n1^{v}	140.93
H1n1—N1—H1n1^{iii}	83.62	H2n1^{iii}—N1—H3n1^{vi}	86.43
H1n1—N1—H1n1^{iv}	80.79	H2n1^{iii}—N1—H3n1^{vii}	80.79
H1n1—N1—H1n1^{v}	86.43	H2n1^{iv}—N1—H2n1^{v}	141.06
H1n1—N1—H1n1^{vi}	2.97	H2n1^{iv}—N1—H2n1^{vi}	83.62
H1n1—N1—H1n1^{vii}	140.93	H2n1^{iv}—N1—H2n1^{vii}	83.62
H1n1—N1—H2n1	109.47	H2n1^{iv}—N1—H3n1	80.79
H1n1—N1—H2n1^{i}	56.25	H2n1^{iv}—N1—H3n1^{i}	86.43
H1n1—N1—H2n1^{ii}	28.25	H2n1^{iv}—N1—H3n1^{ii}	140.93
H1n1—N1—H2n1^{iii}	131.2	H2n1^{iv}—N1—H3n1^{iii}	2.97
H1n1—N1—H2n1^{iv}	31.21	H2n1^{iv}—N1—H3n1^{iv}	109.47
H1n1—N1—H2n1^{v}	129.28	H2n1^{iv}—N1—H3n1^{v}	56.25
H1n1—N1—H2n1^{vi}	112.03	H2n1^{iv}—N1—H3n1^{vi}	131.2
H1n1—N1—H2n1^{vii}	53.33	H2n1^{iv}—N1—H3n1^{vii}	28.25
H1n1—N1—H3n1	109.47	H2n1^{v}—N1—H2n1^{vi}	83.62
H1n1—N1—H3n1^{i}	56.25	H2n1^{v}—N1—H2n1^{vii}	83.62
H1n1—N1—H3n1^{ii}	131.2	H2n1^{v}—N1—H3n1	86.43

Symmetry codes: (i) *x*, -*y*, -*z*; (ii) *x*, *z*, -*y*; (iii) *x*, -*z*, *y*; (iv) *x*, *y*, -*z*; (v) *x*, -*y*, *z*; (vi) *x*, -*z*, -*y*; (vii) *x*, *z*, *y*.

8) Structural Model for $Ni(NH_3)_2Cl_2$

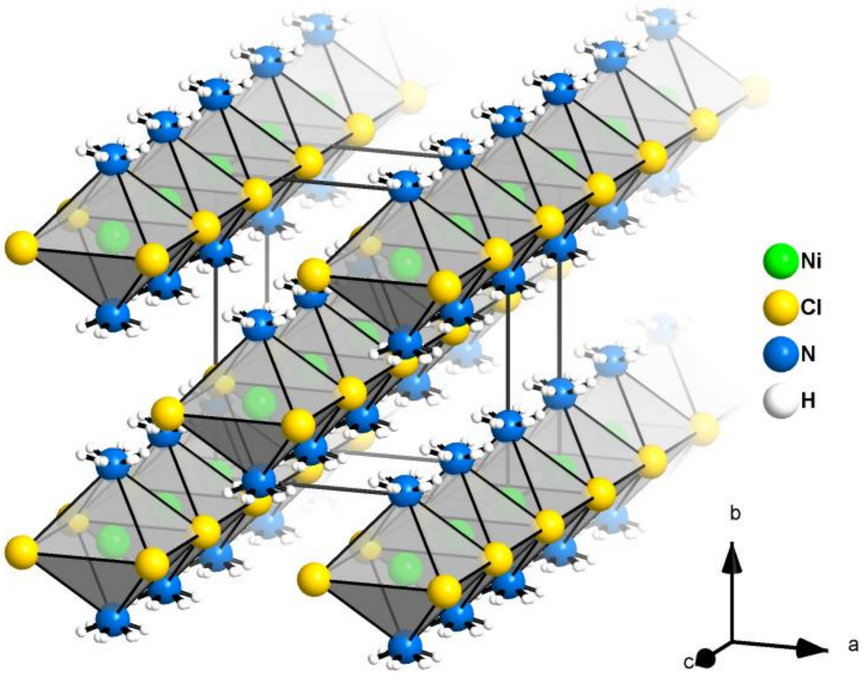

Figure S15: Representation of the structure of $Ni(NH_3)_2Cl_2$. The atom ellipsoids (apart from H) represent 90 % spatial probability (which was set to the default values of the refinement programme). The hydrogen atoms are represented as generic spheres.

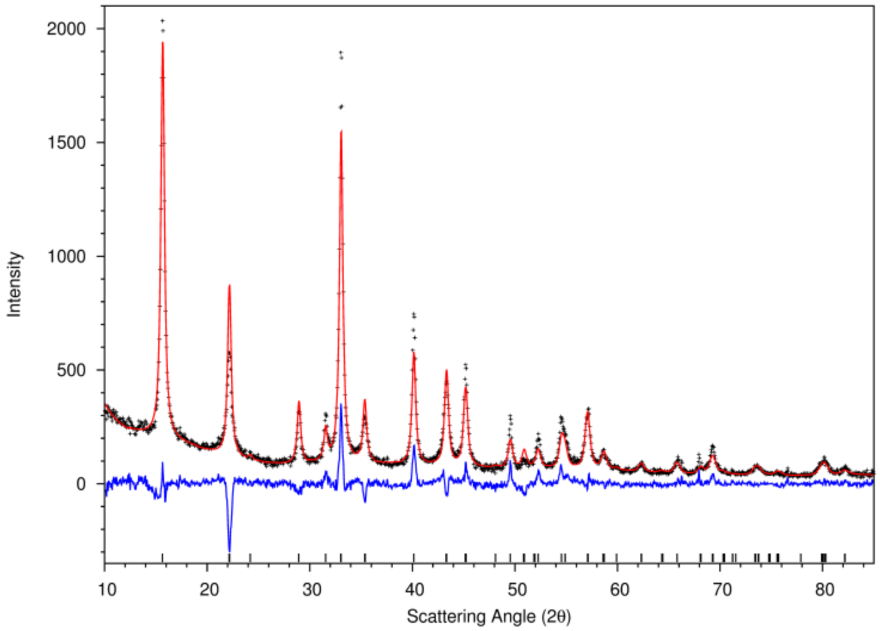

Figure S16: Rietveld refinement profile for $Ni(NH_3)_2Cl_2$. The red line represents the calculated pattern, the black crosses the measured pattern with the difference represented by the blue line. Reflection positions are represented by black ticks.

Table S21: Crystal data

$Cl_2H_6N_2Ni$	*F*(000) = 164
M_r = 163.7	D_x = 2.295 Mg m^{-3}
Orthorhombic, *Cmmm*	Cu $K\alpha_1$ radiation, λ = 1.54056 Å
Hall symbol: -C 2 2	*T* = 373 K
a = 8.016 (3) Å	Particle morphology: irregular
b = 8.030 (3) Å	yellow
c = 3.6779 (16) Å	flat sheet, 5 × 5 mm
V = 236.73 (17) Å^3	Specimen preparation: Prepared at 373 K and ambient kPa
Z = 2	

Table S22: Data collection parameters

Reflection Bragg-Brentano geometry diffractometer	Data collection mode: reflection
Radiation source: sealed X-ray tube, PANalytical XPert Pro	Scan method: continuous
Ge 111 monochromator	$2\theta_{min}$ = 10.04°, $2\theta_{max}$ = 84.99°, $2\theta_{step}$ = 0.05°
Specimen mounting: powder on non-ambient stage with Ar flow, Kapton windows and ceramic sample holder	

Table S23: Refinement parameters

R_p = 0.100	0 restraints
R_{wp} = 0.134	0 constraints
R_{exp} = 0.080	H-atom parameters constrained
R(*F*) = 0.088	Weighting scheme based on measured s.u.'s
χ^2 = 2.789	$(\Delta/\sigma)_{max}$ = 1.328
1500 data points	Background function: 12 Legendre polynoms
Profile function: Pseudo-Voigt	Preferred orientation correction: none
24 parameters	

Table S24: Fractional atomic coordinates and isotropic or equivalent isotropic displacement parameters /Å^2. Asterisks signify isotropically refined thermal displacement parameters (U_{iso}), otherwise U_{eq} signifies the equivalent parameter in the anisotropically refined case.

	x	*y*	*z*	U_{iso}*/U_{eq}	Occ. (<1)
Ni1	0	0	0	0.0317*	
Cl1	0.2226 (7)	0	0.5	0.0317*	
N1	0	0.2541 (14)	0	0.0317*	
H1n1	0.084453	0.290217	0.125921	0.038*	0.25
H2n1	0.007808	0.290217	-0.222366	0.038*	0.25
H3n1	-0.092261	0.290217	0.096445	0.038*	0.25

Table S25: Selected Geometric parameters /Å, º

Ni1—Cl1^{i}	2.562 (4)	N1—H2n1	0.87
Ni1—Cl1	2.562 (4)	N1—H2n1^{x}	0.87
Ni1—Cl1^{ii}	2.562 (4)	N1—H2n1^{xi}	0.87
Ni1—Cl1^{iii}	2.562 (4)	N1—H2n1^{xii}	0.87
Ni1—N1	2.040 (11)	N1—H3n1	0.87
Ni1—N1^{iii}	2.040 (11)	N1—H3n1^{x}	0.87
Cl1—N1	3.275 (8)	N1—H3n1^{xi}	0.87
Cl1—N1^{iv}	3.275 (8)	N1—H3n1^{xii}	0.87
Cl1—N1^{v}	3.497 (8)	H1n1—H1n1^{xi}	0.9263
Cl1—N1^{vi}	3.497 (8)	H1n1—H2n1^{x}	0.8202
Cl1—N1^{iii}	3.275 (8)	H1n1—H2n1^{xi}	0.7094
Cl1—N1^{vii}	3.275 (8)	H1n1—H3n1^{x}	0.8202
Cl1—N1^{viii}	3.497 (8)	H1n1—H3n1^{xii}	0.1252
Cl1—N1^{ix}	3.497 (8)	H2n1—H2n1^{xii}	0.1252
N1—H1n1	0.87	H2n1—H3n1^{x}	0.8202
N1—H1n1^{x}	0.87	H2n1—H3n1^{xi}	0.9263
N1—H1n1^{xi}	0.87	H3n1—H3n1^{xi}	0.7094
N1—H1n1^{xii}	0.87		
Cl1^{i}—Ni1—Cl1	91.74 (14)	Cl1^{ii}—N1—H2n1^{x}	134.13
Cl1^{i}—Ni1—Cl1^{ii}	88.26 (14)	Cl1^{ii}—N1—H2n1^{xi}	140.77
Cl1^{i}—Ni1—Cl1^{iii}	180.0 (5)	Cl1^{ii}—N1—H2n1^{xii}	68.94
Cl1^{i}—Ni1—N1	90	Cl1^{ii}—N1—H3n1	88.48
Cl1^{i}—Ni1—N1^{iii}	90	Cl1^{ii}—N1—H3n1^{x}	116.22
Cl1—Ni1—Cl1^{ii}	180.0 (5)	Cl1^{ii}—N1—H3n1^{xi}	61.03
Cl1—Ni1—Cl1^{iii}	88.26 (14)	Cl1^{ii}—N1—H3n1^{xii}	154.11
Cl1—Ni1—N1	90	Cl1^{iii}—N1—Cl1^{xv}	173.5 (2)
Cl1—Ni1—N1^{iii}	90	Cl1^{iii}—N1—Cl1^{viii}	113.76 (6)
Cl1^{ii}—Ni1—Cl1^{iii}	91.74 (14)	Cl1^{iii}—N1—H1n1	109.43
Cl1^{ii}—Ni1—N1	90	Cl1^{iii}—N1—H1n1^{x}	94.74
Cl1^{ii}—Ni1—N1^{iii}	90	Cl1^{iii}—N1—H1n1^{xi}	158.5
Cl1^{iii}—Ni1—N1	90	Cl1^{iii}—N1—H1n1^{xii}	59
Cl1^{iii}—Ni1—N1^{iii}	90	Cl1^{iii}—N1—H2n1	140.77
N1—Ni1—N1^{iii}	180.0 (5)	Cl1^{iii}—N1—H2n1^{x}	68.94
Ni1—Cl1—Ni1^{iv}	91.74 (19)	Cl1^{iii}—N1—H2n1^{xi}	73.68
Ni1—Cl1—N1	38.53 (16)	Cl1^{iii}—N1—H2n1^{xii}	134.13
Ni1—Cl1—N1^{iv}	91.36 (15)	Cl1^{iii}—N1—H3n1	61.03
Ni1—Cl1—N1^{v}	93.75 (3)	Cl1^{iii}—N1—H3n1^{x}	154.11
Ni1—Cl1—N1^{vi}	145.12 (15)	Cl1^{iii}—N1—H3n1^{xi}	88.48
Ni1—Cl1—N1^{iii}	38.53 (16)	Cl1^{iii}—N1—H3n1^{xii}	116.22
Ni1—Cl1—N1^{vii}	91.36 (15)	Cl1^{xv}—N1—Cl1^{viii}	63.46 (14)
Ni1—Cl1—N1^{viii}	93.75 (3)	Cl1^{xv}—N1—H1n1	66.22

Symmetry codes: (i) *x*, *y*, *z*-1; (ii) -*x*, -*y*, *z*-1; (iii) -*x*, -*y*, *z*; (iv) *x*, *y*, *z*+1; (v) *x*+1/2, *y*-1/2, *z*; (vi) *x*+1/2, *y*-1/2, *z*+1; (vii) -*x*, -*y*, *z*+1; (viii) -*x*+1/2, -*y*+1/2, *z*; (ix) -*x*+1/2, -*y*+1/2, *z*+1; (x) -*x*, *y*, -*z*; (xi) *x*, *y*, -*z*; (xii) -*x*, *y*, *z*; (xiii) *x*-1/2, *y*+1/2, *z*-1; (xiv) *x*-1/2, *y*+1/2, *z*; (xv) -*x*+1/2, -*y*+1/2, *z*-1.

9) Crystallographic Data for $Ni(NH_3)_2Br_2$

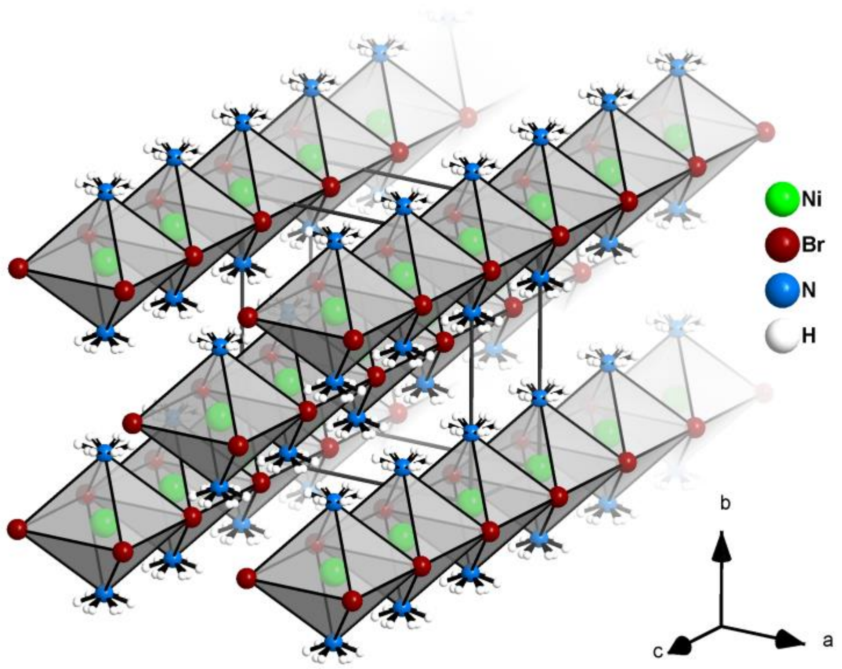

Figure S17: Representation of the structure of $Ni(NH_3)_2Br_2$. The atom ellipsoids (apart from H) represent 90 % spatial probability. The hydrogen atoms are represented as generic spheres.

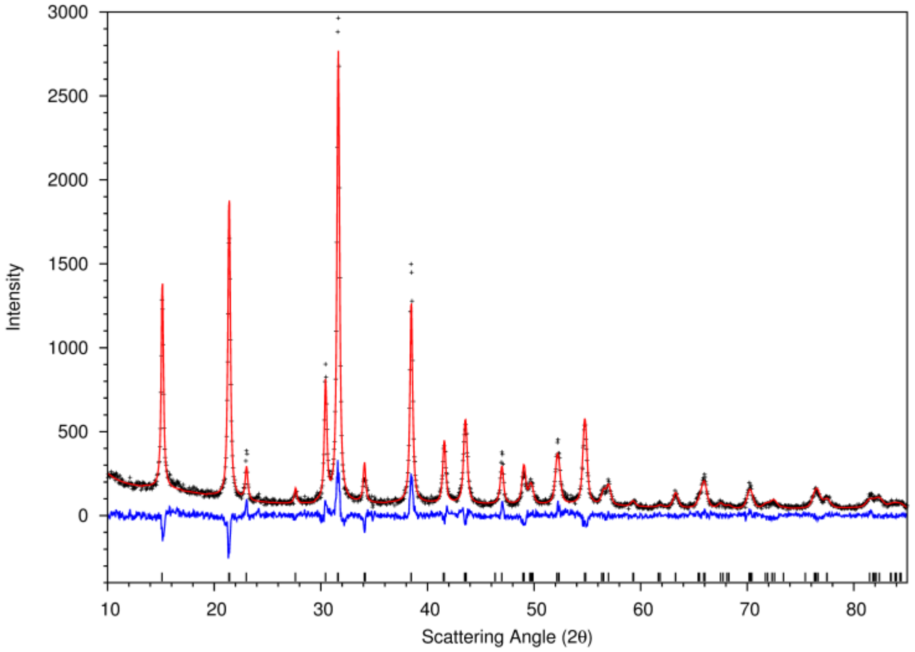

Figure S18: Rietveld refinement profile for $Ni(NH_3)_2Br_2$. The red line represents the calculated pattern, the black crosses the measured pattern with the difference represented by the blue line. Reflection positions are represented by black ticks.

Table S26: Crystal data

$Br_2H_6N_2Ni$	$F(000) = 236$
$M_r = 252.6$	$D_x = 3.138$ Mg m^{-3}
Orthorhombic, *Cmmm*	Cu $K\alpha_1$ radiation, $\lambda = 1.54056$ Å
Hall symbol: -C 2 2	$T = 373$ K
$a = 8.2980$ (16) Å	Particle morphology: irregular
$b = 8.3240$ (15) Å	yellow
$c = 3.8684$ (7) Å	flat sheet, 5 × 5 mm
$V = 267.20$ (9) Å^3	Specimen preparation: Prepared at 373 K and ambient kPa
$Z = 2$	

Table S27: Data collection parameters

Reflection Bragg-Brentano geometry diffractometer	Data collection mode: reflection
Radiation source: sealed X-ray tube, PANalytical XPert Pro	Scan method: continuous
Ge 111 monochromator	$2\theta_{min} = 10.04°$, $2\theta_{max} = 84.98°$, $2\theta_{step} = 0.05°$
Specimen mounting: powder on non-ambient stage with Ar flow, Kapton windows and ceramic sample holder	

Table S28: Refinement parameters

R_p = 0.104	0 restraints
R_{wp} = 0.126	0 constraints
R_{exp} = 0.081	H-atom parameters constrained
$R(F)$ = 0.058	Weighting scheme based on measured s.u.'s
χ^2 = 2.434	$(\Delta/\sigma)_{max}$ = 0.086
1496 data points	Background function: 12 Legendre polynoms
Profile function: Pseudo-Voigt	Preferred orientation correction: none
28 parameters	

Table S29: Fractional atomic coordinates and isotropic or equivalent isotropic displacement parameters /Å^2. Asterisks signify isotropically refined thermal displacement parameters (U_{iso}), otherwise U_{eq} signifies the equivalent parameter in the anisotropically refined case.

	x	*y*	*z*	U_{iso}*/U_{eq}	Occ. (<1)
Ni1	0	0	0	0.032 (4)*	
Br1	0.2298 (4)	0	0.5	0.0168 (17)*	
N1	0	0.2677 (18)	0	0.013 (6)*	
H1n1	-0.011118	0.302569	0.210693	0.0151*	0.25
H2n1	0.090621	0.302569	-0.084693	0.0151*	0.25
H3n1	-0.079504	0.302569	-0.125999	0.0151*	0.25

Table S30: Selected Geometric parameters / Å, º

Ni1—Br1^{i}	2.716 (3)	N1—H2n1	0.87
Ni1—Br1	2.716 (3)	N1—H2n1^{x}	0.87
Ni1—Br1^{ii}	2.716 (3)	N1—H2n1^{xi}	0.87
Ni1—Br1^{iii}	2.716 (3)	N1—H2n1^{xii}	0.87
Ni1—N1	2.229 (15)	N1—H3n1	0.87
Ni1—N1^{iii}	2.229 (15)	N1—H3n1^{x}	0.87
Br1—N1	3.513 (10)	N1—H3n1^{xi}	0.87
Br1—N1^{iv}	3.513 (10)	N1—H3n1^{xii}	0.87
Br1—N1^{v}	3.537 (9)	H1n1—H1n1^{xii}	0.1845
Br1—N1^{vi}	3.537 (9)	H1n1—H2n1^{x}	0.8202
Br1—N1^{iii}	3.513 (10)	H1n1—H2n1^{xi}	0.9748
Br1—N1^{vii}	3.513 (10)	H1n1—H3n1^{x}	0.8203
Br1—N1^{viii}	3.537 (9)	H1n1—H3n1^{xi}	0.6553
Br1—N1^{ix}	3.537 (9)	H2n1—H2n1^{xi}	0.6552
N1—H1n1	0.87	H2n1—H3n1^{x}	0.8202
N1—H1n1^{x}	0.87	H2n1—H3n1^{xii}	0.1845
N1—H1n1^{xi}	0.87	H3n1—H3n1^{xi}	0.9748
N1—H1n1^{xii}	0.87		
Br1^{i}—Ni1—Br1	90.82 (8)	Br1^{ii}—N1—H2n1^{x}	87.12
Br1^{i}—Ni1—Br1^{ii}	89.18 (8)	Br1^{ii}—N1—H2n1^{xi}	152.6
Br1^{i}—Ni1—Br1^{iii}	180.0 (5)	Br1^{ii}—N1—H2n1^{xii}	62.29
Br1^{i}—Ni1—N1	90	Br1^{ii}—N1—H3n1	59.44
Br1^{i}—Ni1—N1^{iii}	90	Br1^{ii}—N1—H3n1^{x}	158.65
Br1—Ni1—Br1^{ii}	180.0 (5)	Br1^{ii}—N1—H3n1^{xi}	96.22
Br1—Ni1—Br1^{iii}	89.18 (8)	Br1^{ii}—N1—H3n1^{xii}	108.33
Br1—Ni1—N1	90	Br1^{iii}—N1—Br1^{xv}	172.7 (3)
Br1—Ni1—N1^{iii}	90	Br1^{iii}—N1—Br1^{viii}	112.94 (5)
Br1^{ii}—Ni1—Br1^{iii}	90.82 (8)	Br1^{iii}—N1—H1n1	68.79
Br1^{ii}—Ni1—N1	90	Br1^{iii}—N1—H1n1^{x}	141.7
Br1^{ii}—Ni1—N1^{iii}	90	Br1^{iii}—N1—H1n1^{xi}	132.04
Br1^{iii}—Ni1—N1	90	Br1^{iii}—N1—H1n1^{xii}	75.71
Br1^{iii}—Ni1—N1^{iii}	90	Br1^{iii}—N1—H2n1	152.6
N1—Ni1—N1^{iii}	180.0 (5)	Br1^{iii}—N1—H2n1^{x}	62.29
Ni1—Br1—Ni1^{iv}	90.82 (11)	Br1^{iii}—N1—H2n1^{xi}	118.24
Ni1—Br1—N1	39.37 (19)	Br1^{iii}—N1—H2n1^{xii}	87.12
Ni1—Br1—N1^{iv}	90.64 (9)	Br1^{iii}—N1—H3n1	96.22
Ni1—Br1—N1^{v}	93.188 (16)	Br1^{iii}—N1—H3n1^{x}	108.33
Ni1—Br1—N1^{vi}	146.6 (2)	Br1^{iii}—N1—H3n1^{xi}	59.44
Ni1—Br1—N1^{iii}	39.37 (19)	Br1^{iii}—N1—H3n1^{xii}	158.65
Ni1—Br1—N1^{vii}	90.64 (9)	Br1^{xv}—N1—Br1^{viii}	66.31 (18)
Ni1—Br1—N1^{viii}	93.188 (16)	Br1^{xv}—N1—H1n1	113.41
Ni1—Br1—N1^{ix}	146.6 (2)	Br1^{xv}—N1—H1n1^{x}	40.38
Ni1^{iv}—Br1—N1	90.64 (9)	Br1^{xv}—N1—H1n1^{xi}	51.15

$Ni1^{iv}$—Br1—$N1^{iv}$	39.37 (19)	$Br1^{xv}$—N1—$H1n1^{xii}$	105.24
$Ni1^{iv}$—Br1—$N1^{v}$	146.6 (2)	$Br1^{xv}$—N1—H2n1	20.57
$Ni1^{iv}$—Br1—$N1^{vi}$	93.188 (16)	$Br1^{xv}$—N1—$H2n1^{x}$	124.87
$Ni1^{iv}$—Br1—$N1^{iii}$	90.64 (9)	$Br1^{xv}$—N1—$H2n1^{xi}$	58.38
$Ni1^{iv}$—Br1—$N1^{vii}$	39.37 (19)	$Br1^{xv}$—N1—$H2n1^{xii}$	99.2
$Ni1^{iv}$—Br1—$N1^{viii}$	146.6 (2)	$Br1^{xv}$—N1—H3n1	89.55
$Ni1^{iv}$—Br1—$N1^{ix}$	93.188 (16)	$Br1^{xv}$—N1—$H3n1^{x}$	69.11
N1—Br1—$N1^{iv}$	66.81 (15)	$Br1^{xv}$—N1—$H3n1^{xi}$	127.23
N1—Br1—$N1^{v}$	112.94 (14)	$Br1^{xv}$—N1—$H3n1^{xii}$	14.2
N1—Br1—$N1^{vi}$	172.7 (3)	$Br1^{viii}$—N1—H1n1	51.15
N1—Br1—$N1^{iii}$	78.7 (3)	$Br1^{viii}$—N1—$H1n1^{x}$	105.24

Symmetry codes: (i) *x*, *y*, *z*-1; (ii) -*x*, -*y*, *z*-1; (iii) -*x*, -*y*, *z*; (iv) *x*, *y*, *z*+1; (v) *x*+1/2, *y*-1/2, *z*; (vi) *x*+1/2, *y*-1/2, *z*+1; (vii) -*x*, -*y*, *z*+1; (viii) -*x*+1/2, -*y*+1/2, *z*; (ix) -*x*+1/2, -*y*+1/2, *z*+1; (x) -*x*, *y*, -*z*; (xi) *x*, *y*, -*z*; (xii) -*x*, *y*, *z*; (xiii) *x*-1/2, *y*+1/2, *z*-1; (xiv) *x*-1/2, *y*+1/2, *z*; (xv) -*x*+1/2, -*y*+1/2, *z*-1.

10) Crystallographic Data for $Ni(NH_3)_2I_2$

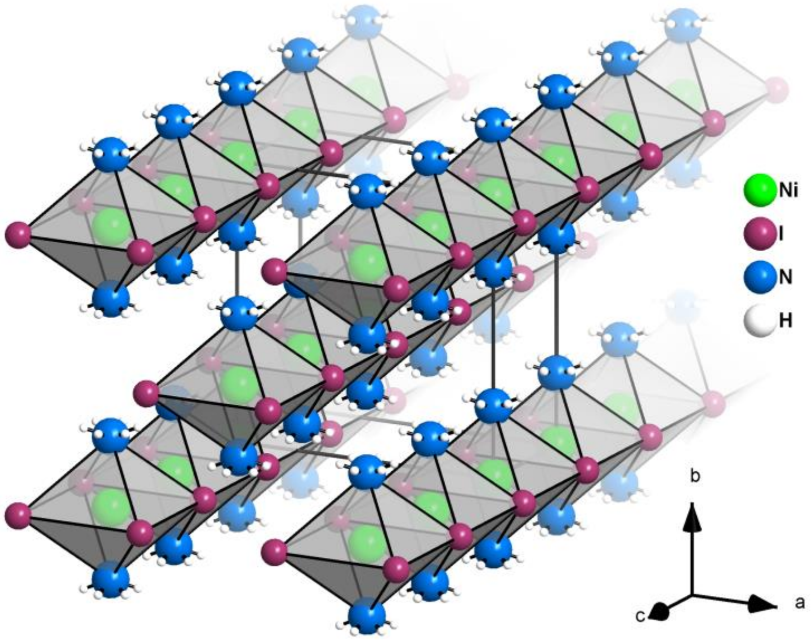

Figure S19: Representation of the structure of $Ni(NH_3)_2I_2$. The atom ellipsoids (apart from H) represent 90 % spatial probability. The hydrogen atoms are represented as generic spheres.

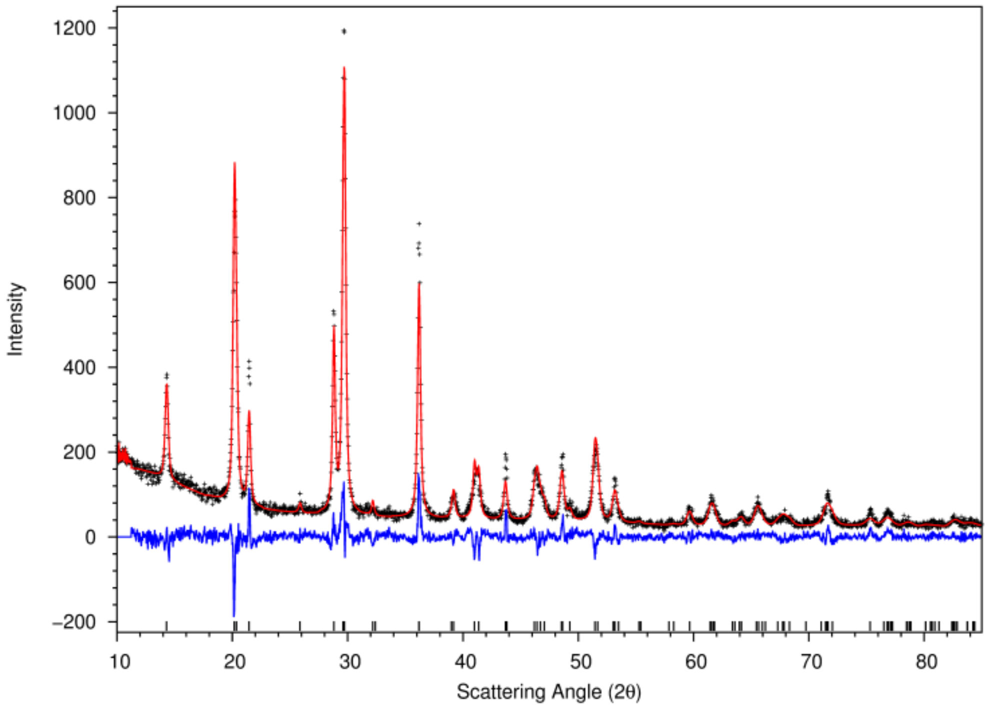

Figure S20: Rietveld refinement profile for $Ni(NH_3)_2I_2$. The red line represents the calculated pattern, the black crosses the measured pattern with the difference represented by the blue line. Reflection positions are represented by black ticks.

Table S31: Crystal data

$H_6I_2N_2Ni$	$F(000) = 308$
$M_r = 346.6$	$D_x = 3.618$ Mg m^{-3}
Orthorhombic, *Cmmm*	Cu $K\alpha_1$ radiation, $\lambda = 1.54056$ Å
Hall symbol: -C 2 2	$T = 393$ K
$a = 8.726$ (3) Å	Particle morphology: irregular
$b = 8.800$ (2) Å	yellow
$c = 4.1416$ (12) Å	flat sheet, 5 × 5 mm
$V = 318.02$ (16) Å^3	Specimen preparation: Prepared at 393 K and ambient kPa
$Z = 2$	

Table S32: Data collection parameters

Reflection Bragg-Brentano geometry diffractometer	Data collection mode: reflection
Radiation source: sealed X-ray tube, PANalytical XPert Pro	Scan method: continuous
Ge 111 monochromator	$2\theta_{min} = 11.26°$, $2\theta_{max} = 84.98°$, $2\theta_{step} = 0.03°$
Specimen mounting: powder on non-ambient stage with Ar flow, Kapton windows and ceramic sample holder	

Table S33: Refinement parameters

$R_p = 0.105$	28 parameters
$R_{wp} = 0.134$	0 restraints
$R_{exp} = 0.110$	0 constraints
$R(F) = 0.073$	H-atom parameters constrained
$\chi^2 = 1.464$	Weighting scheme based on measured s.u.'s
2424 data points	$(\Delta/\sigma)_{max} = 0.044$
Excluded region(s): from 5.02 to 11.243	Background function: 12 Legendre polynoms
Profile function: Pseudo-Voigt	Preferred orientation correction: none

Table S34: Fractional atomic coordinates and isotropic or equivalent isotropic displacement parameters /Å^2. Asterisks signify isotropically refined thermal displacement parameters (U_{iso}), otherwise U_{eq} signifies the equivalent parameter in the anisotropically refined case.

	x	*y*	*z*	U_{iso}*/U_{eq}	Occ. (<1)
Ni1	0	0	0	0.056 (6)*	
I1	0.2374 (5)	0	0.5	0.028 (2)*	
N1	0	0.254 (4)	0	0.053 (9)*	
H1n1	-0.000671	0.28655	0.198043	0.0639*	0.25
H2n1	0.081745	0.28655	-0.097797	0.0639*	0.25
H3n1	-0.081073	0.28655	-0.100246	0.0639*	0.25

Table S35: Geometric parameters /Å, º

Ni1—I1^{i}	2.929 (3)	N1—H2n1	0.87
Ni1—I1	2.929 (3)	N1—H2n1^{x}	0.87
Ni1—I1^{ii}	2.929 (3)	N1—H2n1^{xi}	0.87
Ni1—I1^{iii}	2.929 (3)	N1—H2n1^{xii}	0.87
Ni1—N1	2.23 (3)	N1—H3n1	0.87
Ni1—N1^{iii}	2.23 (3)	N1—H3n1^{x}	0.87
I1—N1	3.68 (2)	N1—H3n1^{xi}	0.87
I1—N1^{iv}	3.68 (2)	N1—H3n1^{xii}	0.87
I1—N1^{v}	3.774 (19)	H1n1—H1n1^{xii}	0.0117
I1—N1^{vi}	3.774 (19)	H1n1—H2n1^{x}	0.8202
I1—N1^{iii}	3.68 (2)	H1n1—H2n1^{xi}	0.8304
I1—N1^{vii}	3.68 (2)	H1n1—H3n1^{x}	0.8202
I1—N1^{viii}	3.774 (19)	H1n1—H3n1^{xi}	0.8101
I1—N1^{ix}	3.774 (19)	H2n1—H2n1^{xi}	0.8101
N1—H1n1	0.87	H2n1—H3n1^{x}	0.8202
N1—H1n1^{x}	0.87	H2n1—H3n1^{xii}	0.0117
N1—H1n1^{xi}	0.87	H3n1—H3n1^{xi}	0.8304
N1—H1n1^{xii}	0.87		
I1^{i}—Ni1—I1	89.99 (9)	I1^{ii}—N1—H2n1^{x}	90.15
I1^{i}—Ni1—I1^{ii}	90.01 (9)	I1^{ii}—N1—H2n1^{xi}	157.67
I1^{i}—Ni1—I1^{iii}	180.0 (5)	I1^{ii}—N1—H2n1^{xii}	58.6
I1^{i}—Ni1—N1	90	I1^{ii}—N1—H3n1	58.42
I1^{i}—Ni1—N1^{iii}	90	I1^{ii}—N1—H3n1^{x}	158.09
I1—Ni1—I1^{ii}	180.0 (5)	I1^{ii}—N1—H3n1^{xi}	90.75
I1—Ni1—I1^{iii}	90.01 (9)	I1^{ii}—N1—H3n1^{xii}	113.02
I1—Ni1—N1	90	I1^{iii}—N1—I1^{xv}	176.8 (6)
I1—Ni1—N1^{iii}	90	I1^{iii}—N1—I1^{viii}	112.41 (5)
I1^{ii}—Ni1—I1^{iii}	89.99 (9)	I1^{iii}—N1—H1n1	70.61
I1^{ii}—Ni1—N1	90	I1^{iii}—N1—H1n1^{x}	137.39
I1^{ii}—Ni1—N1^{iii}	90	I1^{iii}—N1—H1n1^{xi}	136.76
I1^{iii}—Ni1—N1	90	I1^{iii}—N1—H1n1^{xii}	71.07
I1^{iii}—Ni1—N1^{iii}	90	I1^{iii}—N1—H2n1	157.67
N1—Ni1—N1^{iii}	180.0 (5)	I1^{iii}—N1—H2n1^{x}	58.6
Ni1—I1—Ni1^{iv}	89.99 (13)	I1^{iii}—N1—H2n1^{xi}	113.66
Ni1—I1—N1	37.3 (4)	I1^{iii}—N1—H2n1^{xii}	90.15
Ni1—I1—N1^{iv}	89.99 (10)	I1^{iii}—N1—H3n1	90.75
Ni1—I1—N1^{v}	92.37 (2)	I1^{iii}—N1—H3n1^{x}	113.02
Ni1—I1—N1^{vi}	144.8 (4)	I1^{iii}—N1—H3n1^{xi}	58.42
Ni1—I1—N1^{iii}	37.3 (4)	I1^{iii}—N1—H3n1^{xii}	158.09
Ni1—I1—N1^{vii}	89.99 (10)	I1^{xv}—N1—I1^{viii}	66.6 (4)
Ni1—I1—N1^{viii}	92.37 (2)	I1^{xv}—N1—H1n1	109.26
Ni1—I1—N1^{ix}	144.8 (4)	I1^{xv}—N1—H1n1^{x}	44.52

Ni1^{iv}—I1—N1	89.99 (10)	I1^{xv}—N1—H1n1^{xi}	45.19
Ni1^{iv}—I1—N1^{iv}	37.3 (4)	I1^{xv}—N1—H1n1^{xii}	108.77
Ni1^{iv}—I1—N1^{v}	144.8 (4)	I1^{xv}—N1—H2n1	19.12
Ni1^{iv}—I1—N1^{vi}	92.37 (2)	I1^{xv}—N1—H2n1^{x}	124.18
Ni1^{iv}—I1—N1^{iii}	89.99 (10)	I1^{xv}—N1—H2n1^{xi}	64.29
Ni1^{iv}—I1—N1^{vii}	37.3 (4)	I1^{xv}—N1—H2n1^{xii}	92.91
Ni1^{iv}—I1—N1^{viii}	144.8 (4)	I1^{xv}—N1—H3n1	92.31
Ni1^{iv}—I1—N1^{ix}	92.37 (2)	I1^{xv}—N1—H3n1^{x}	64.95
N1—I1—N1^{iv}	68.4 (3)	I1^{xv}—N1—H3n1^{xi}	124.34
N1—I1—N1^{v}	112.4 (3)	I1^{xv}—N1—H3n1^{xii}	18.71
N1—I1—N1^{vi}	176.8 (4)	I1^{viii}—N1—H1n1	45.19
N1—I1—N1^{iii}	74.6 (6)	I1^{viii}—N1—H1n1^{x}	108.77
N1—I1—N1^{vii}	111.5 (3)	I1^{viii}—N1—H1n1^{xi}	109.26

Symmetry codes: (i) *x*, *y*, *z*-1; (ii) -*x*, -*y*, *z*-1; (iii) -*x*, -*y*, *z*; (iv) *x*, *y*, *z*+1; (v) *x*+1/2, *y*-1/2, *z*; (vi) *x*+1/2, *y*-1/2, *z*+1; (vii) -*x*, -*y*, *z*+1; (viii) -*x*+1/2, -*y*+1/2, *z*; (ix) -*x*+1/2, -*y*+1/2, *z*+1; (x) -*x*, *y*, -*z*; (xi) *x*, *y*, -*z*; (xii) -*x*, *y*, *z*; (xiii) *x*-1/2, *y*+1/2, *z*-1; (xiv) *x*-1/2, *y*+1/2, *z*; (xv) -*x*+1/2, -*y*+1/2, *z*-1.

11) Variable temperature *in-situ* PXD of $[Ni(NH_3)_6]Br_2$ and $[Ni(NH_3)_6]I_2$

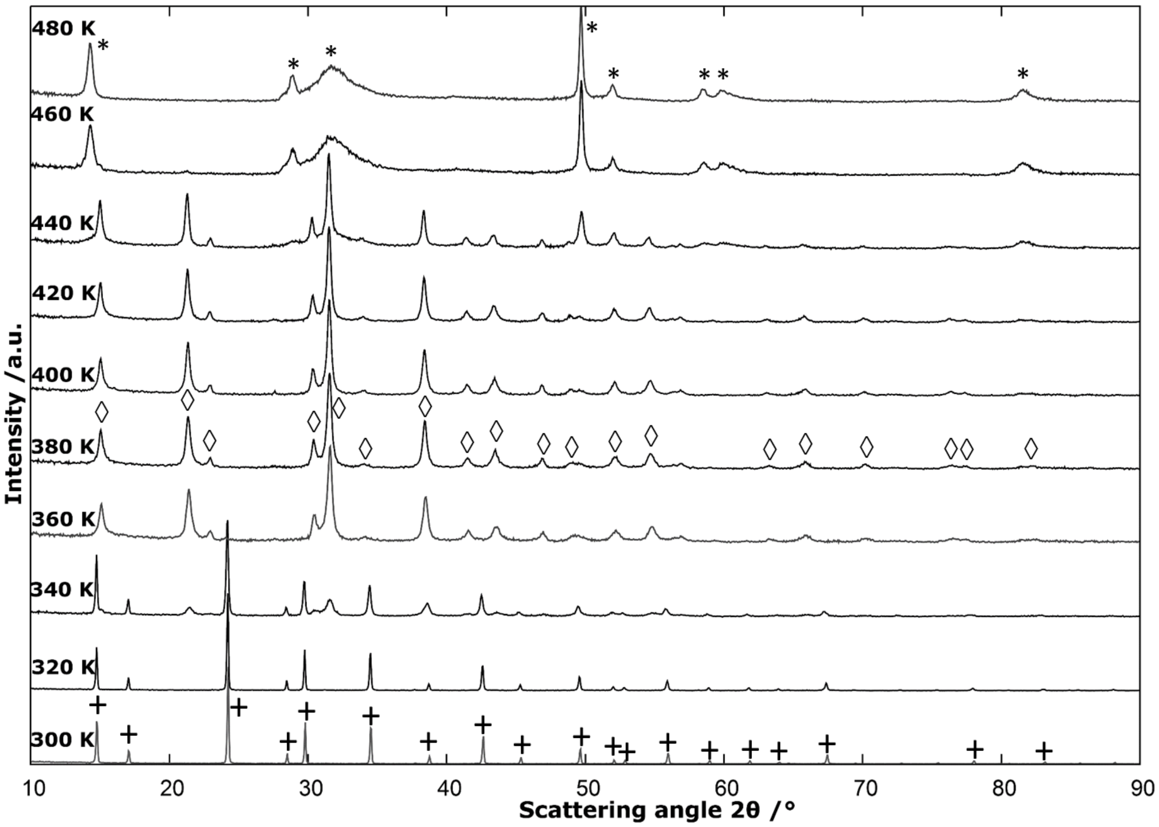

Figure S21: Variable temperature in-situ PXD patterns of the thermal decomposition of $[Ni(NH_3)_6]Br_2$ indicating the presence of the $[Ni(NH_3)_6]Br_2$ (+), $Ni(NH_3)_2Br_2$ (◊) and $NiBr_2$ (*) phases.

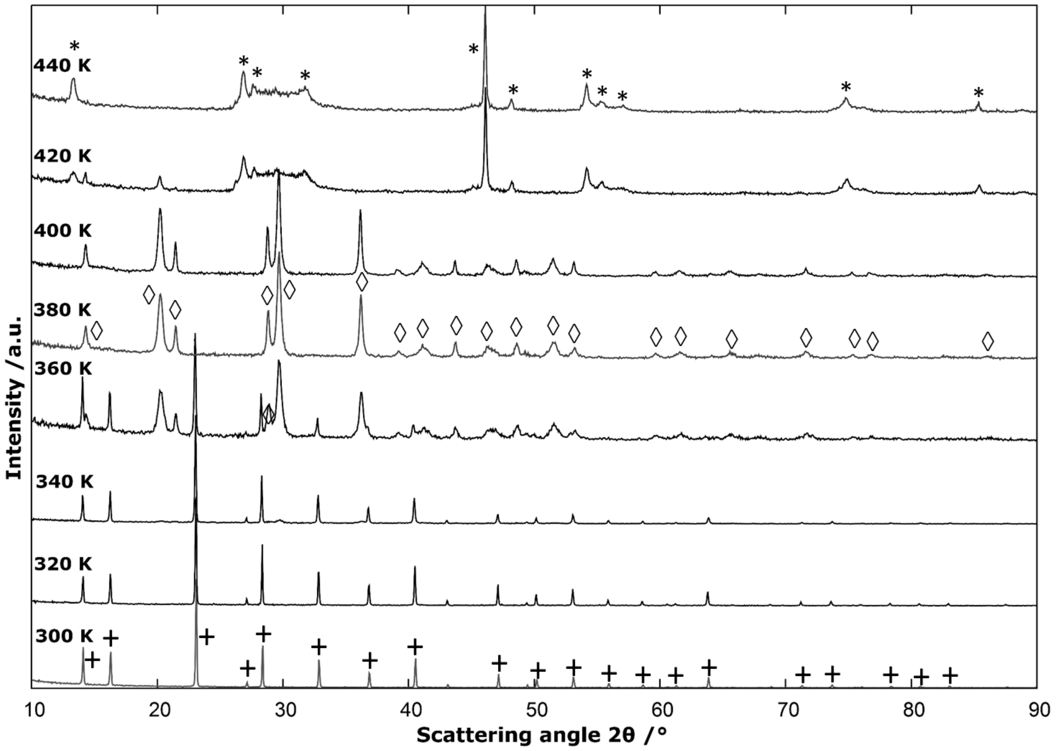

Figure S22: Variable temperature in-situ PXD patterns of the thermal decomposition of $[Ni(NH_3)_6]I_2$ indicating the presence of the $[Ni(NH_3)_6]I_2$ (+), $Ni(NH_3)_2I_2$ (◊) and NiI_2 (*) phases.

12) Cycling experiment

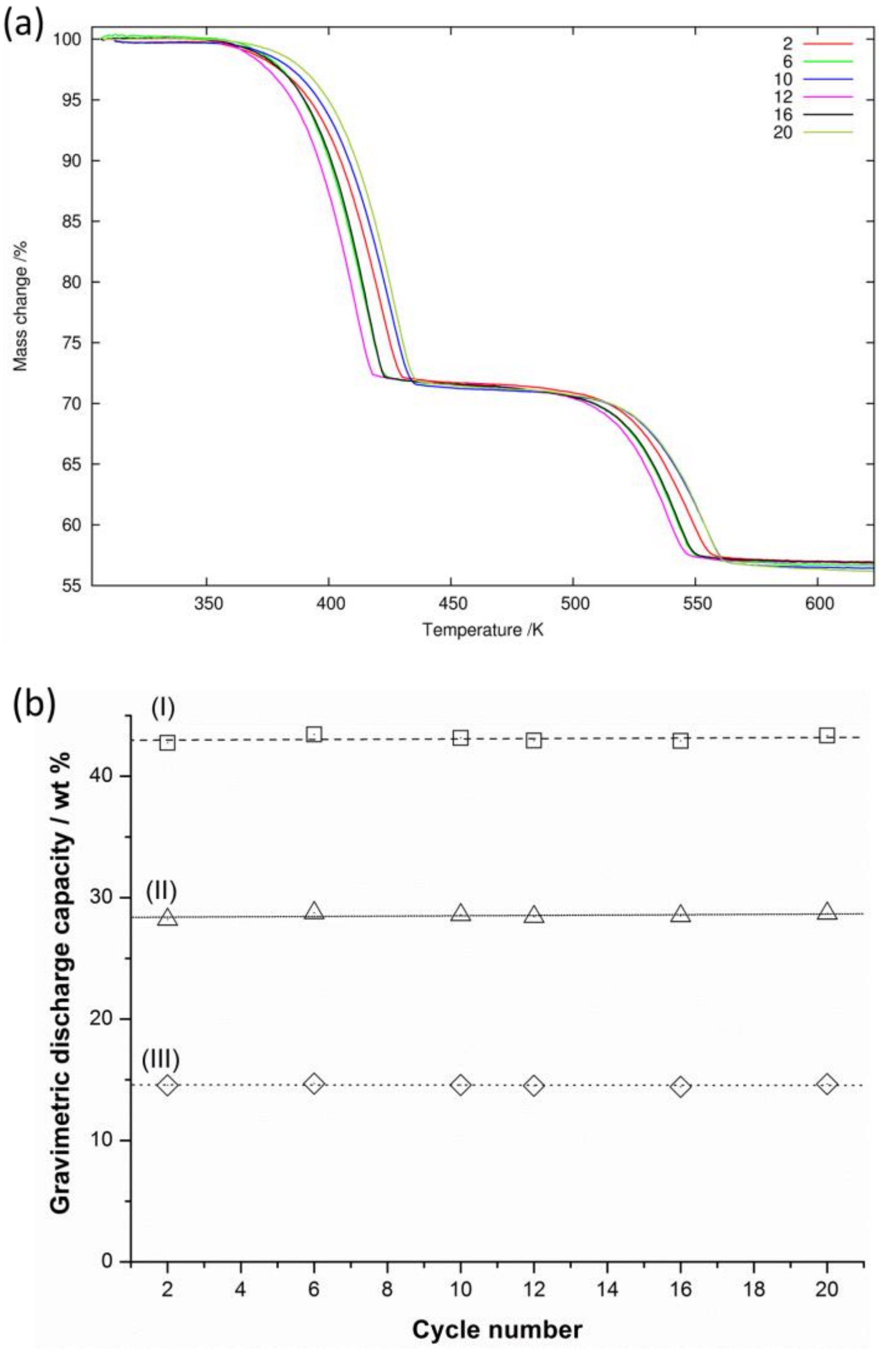

Figure S23 (a) TG curves of the cycled $[Ni(NH_3)_6]Cl_2$ after 2 (red), 6 (green), 10 (blue), 12 (magenta), 16 (black) and 20 (olive) charging cycles; (b) Plot of gravimetric capacity against cycle number indicating the mass losses from n=6 to n=0 (I; open squares) and n=6 to n=2 (II; open triangles) and n=2 to n=0 (III; open diamonds). The dashed, solid and dotted lines represent linear fits to the data.

Table S36: Table of mass losses and deammoniation temperatures for cycled $Ni(NH_3)_6Cl_2$.

Cycle	$n(NH_3)$	Mass loss obs.(calc.) / wt.-%		TGA temperatures / K		DTA peak temperature / K	Sample mass / mg
	final	Cumulative	Stepwise	Onset	Final		
**2**	2	28.2 (29.4)	28.2 (29.4)	385.0	429.0	424.6	11.738
	0	42.75 (44.0)	14.55 (14.6)	524.5	555.3	551.3	
**6**	2	28.75	28.75	384.0	422.5	418.4	8.048
	0	43.44	14.69	519.4	549.4	545.3	
**10**	2	28.58	28.58	390.2	432.9	428.5	15.838
	0	43.15	14.57	524.9	560.5	556.4	
**12**	2	28.43	28.43	382.3	417.1	414.2	6.698
	0	42.94	14.51	520.4	545.3	542.0	
**16**	2	28.49	28.49	385.4	421.8	419.9	8.502
	0	42.91	14.42	521.9	548.6	545.8	
**20**	2	28.7	28.7	390.2	434.0	430.2	15.876
	0	43.35	14.65	528.4	560.5	557.1	

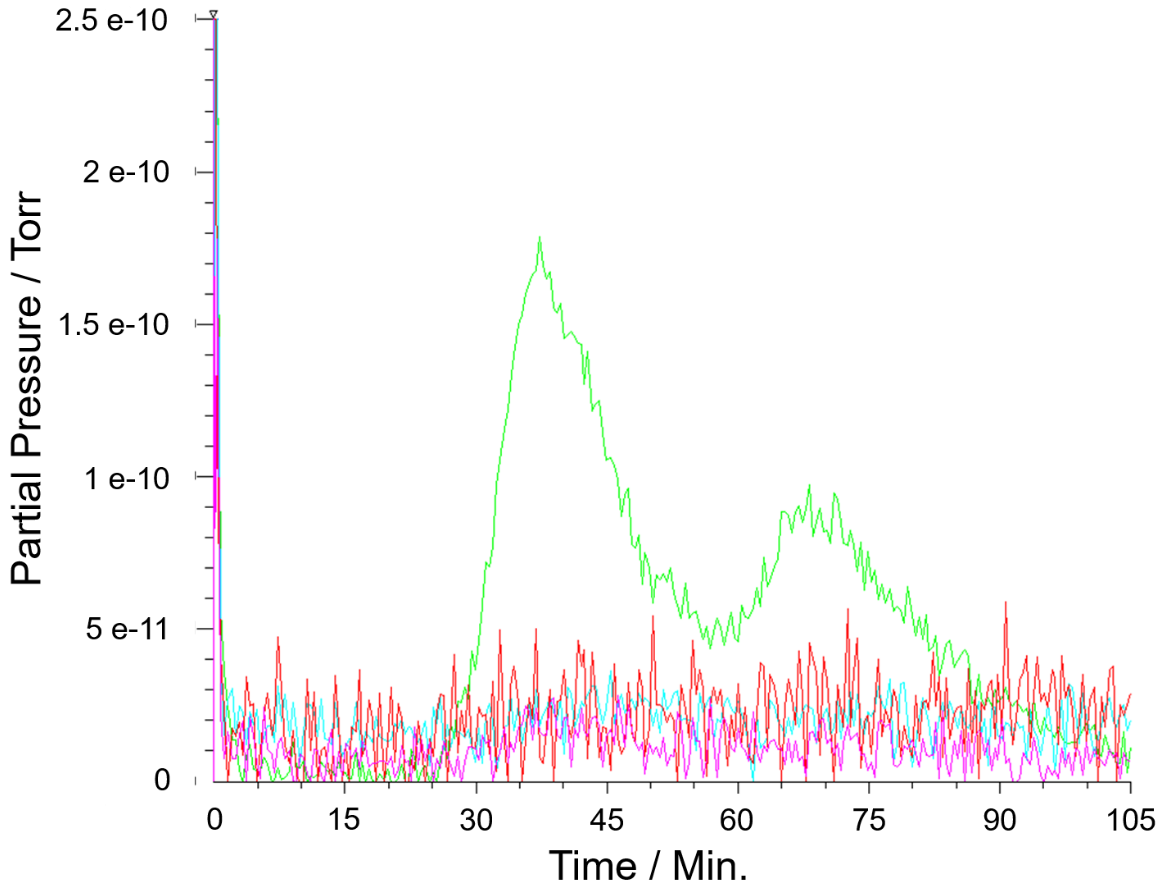

Figure S24: MS trace from the TG-DTA-MS experiment performed on $[Ni(NH_3)_6]Cl_2$ after 20 cycles of (dis)charging with m/z = 2(H_2^+, magenta), 17(NH_3^+, green), 28(N_2^+, red) and 16 (H_2O^+, cyan).

13) Kissinger Analysis

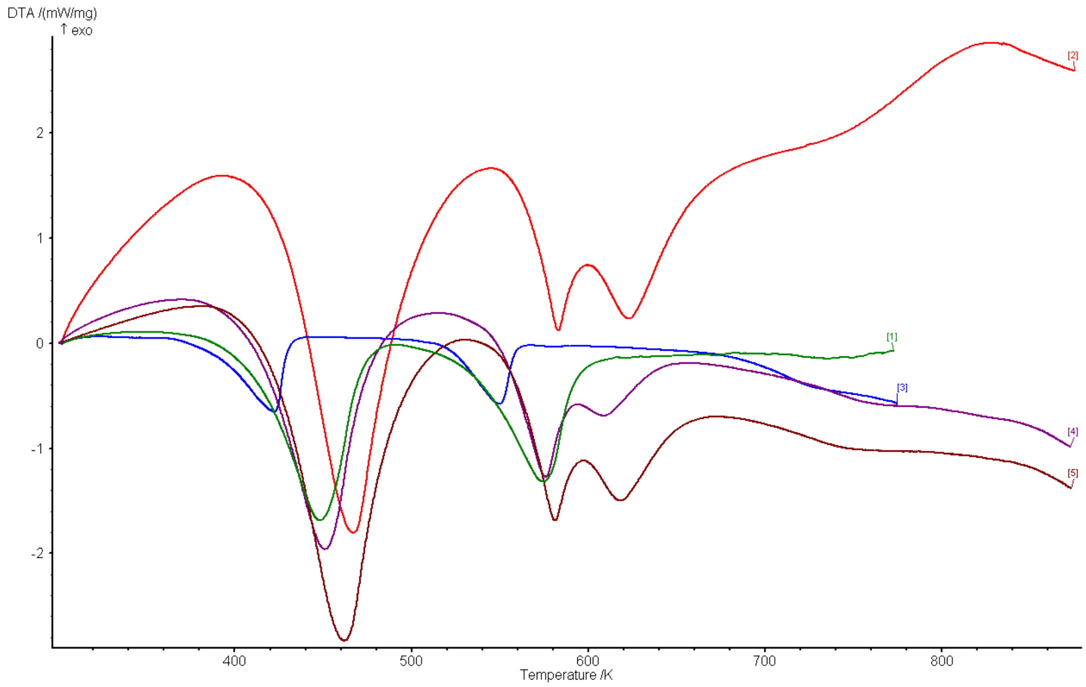

Figure S25: DTA profiles of $[Ni(NH_3)_6]Cl_2$ obtained at different heating rates (blue: 2K min^{-1}, green: 5 K min^{-1}, violet: 10 K min^{-1}, brown: 15 K min^{-1}, red: 20 K min^{-1}).

Table S37: Analysis of the DTA data for $[Ni(NH_3)_6]Cl_2$ obtained at varied heating rates.

Heating rate / K min^{-1}	Peak 1 (hexammine decomposition) / K	Peak 2 (diammine decomposition) / K	Peak 3 (monoammine decomposition) / K
2	422.4	549.9	
5	448.3	574.8	
10	451	576.2	608.7
15	462	581.5	618.8
20	467.1	583.5	623.1

Note: The Kissinger analyses for the higher temperature steps (dihalide diamine/monoamine to dihalide) were solely fitted for those measurements where the steps could be resolved (*i.e.* at 10, 15 and 20 K min^{-1}) so as to avoid ambiguities with the kinetics of the overlapping decomposition processes at lower heating rates (i.e. at 2, and 5 K min^{-1}).

14) Analysis of the hydrogen bonding in $[Ni(NH_3)_6]Cl_2$

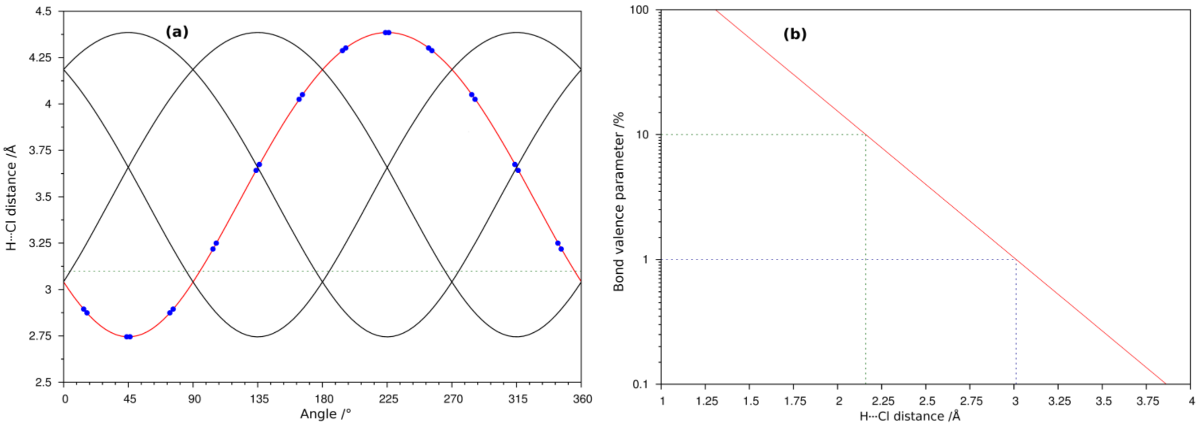

Figure S26 (a) Plot of all possible H···Cl distances between the hydrogen position circle and the four surrounding Cl atoms (red and black lines) as a function of the ammonia rotation angle. The function for one Cl atom is highlighted (red line) and the H···Cl distances used in the refinement are given as blue circles. The horizontal green dashed line marks 3.1 Å, corresponding to a bond valence parameter of ~0.01; **(b)** Plot of the bond-valence parameter as a function of the H···Cl bonding distance (red line). The green and blue dashed lines correspond to the bond lengths representing 10 % and 1 % of covalent bond strength respectively. (N.b. the cut-off distance of 3.1 Å in (a) is slightly longer than the calculated values shown in (b) and takes account for the systematic overestimation of the H···Cl bond lengths (of ~0.1 Å) in X-ray data.)

References:

[S1] A. Müller, K. H. Schmidt, G. Vandrish, *Spectrochim. Acta*, 1974, **A30**, 651.

[S2] R. Eßmann, C. Mockenhaupt, *Spectrochim. Acta*, 1996, **A52**, 1897–1901.
